# Disentangling leaf day respiration from photorespiration in C_3_ leaves: A single-curve *A*_n_*/C*_a_ approach validated under photorespiration-suppressed conditions

**DOI:** 10.1371/journal.pone.0361238

**Published:** 2026-10-23

**Authors:** Zipiao Ye, Xiaolong Yang, Ting An, Zhengwen Niu, Qi Huang, Chunju Peng, Fubiao Wang, Huajing Kang

**Affiliations:** 1 Photosynthesis Research Institute, Nanchang Institute of Technology, Nanchang, Jiangxi, P.R. China; 2 New Quality Productivity Research Center, Guangdong ATV College of Performing Arts, Deqing, P. R. China; 3 Wenzhou Academy of Agricultural Sciences, Wenzhou, Zhejiang, P. R. China; 4 School of Life Sciences, Nantong University, Nantong, China; 5 State Key Laboratory of Environmental Chemistry and Ecotoxicology, Research Center for Eco-Environmental Sciences, Chinese Academy of Sciences, Beijing, China; 6 Key Laboratory of Crop Breeding in South Zhejiang, Wenzhou Academy of Agricultural Sciences, Wenzhou, China; 7 Institute of Biophysics, Math & Physics College, Jinggangshan University, Ji’an, P. R. of China; University of Innsbruck, AUSTRIA

## Abstract

Leaf day respiratory rate (*R*_d_), a pivotal component of plant carbon metabolism that directly modulates net photosynthetic carbon gain and plant fitness, has long been challenging to accurately quantify due to overlapping CO_2_ fluxes with photosynthesis and photorespiration (*R*_p_). This study aimed to disentangle *R*_d_ from *R*_p_ in C_3_ crops using a high-CO_2_/low-O_2_ (>1200 μmol mol^–1^ CO_2_ + 2% O_2_) *A*_n_*/C*_a_ (net photosynthetic rate-ambient CO_2_ concentration) model. We integrated two datasets: *A*_n_*/C*_a_ curve data of four C_3_ species (*Trifolium pratense*, *Silphium perfoliatum*, *Lolium perenne*, *Triticum aestivum*) under ambient O_2_ (21% O_2_) from Ye et al. (2025) and newly collected data under low O_2_ (2% O_2_) for the same species. A dedicated model (Eqn 1) was developed to extract key parameters [e.g., the maximum net photosynthetic rate (*A*_nmax_), CO_2_ compensation point (*Γ*) in the presence of *R*_d_, photorespiratory rate at *C*_a_ = 0 μmol mol^–1^ (*R*_C0_)] from *A*_n_*/C*_a_ curves, with ANOVA confirming no significant differences between calculated and observed parameter values (*p* > 0.05). Under 2% O_2_, *R*_p_ was strongly suppressed, making the gross photorespiratory rate (*R*_C_ = *R*_p_ + *R*_d_) a reliable proxy for approximate *R*_d_. Results showed that *R*_d_ estimates from the high-CO_2_/low-O_2_ approach (0.74–1.12 μmol m^–2^ s^–1^) were consistent with literature ranges (0.74–2.89 μmol m^–2^ s^–1^) for four C_3_ crops. Interspecific differences in *R*_d_ were observed: *L. perenne* had the highest *R*_d_, while *T. aestivum* had the lowest, which correlated with species-specific photosynthetic traits (e.g., carboxylation efficiency). *R*_d_ remained stable across O_2_ levels (21% *vs*. 2%), indicating independence from short-term photorespiratory flux changes. The *R*_C_ ≈ *R*_d_ assumption is supported by the substantial decrease in *Γ* (50–67%) and *R*_CO_ (40–55%) under 2% O_2_, and by the consistency of *R*_d_ estimates with literature ranges, although independent ground-truth measurements remain to be conducted. This study suggests the high-CO_2_/low-O_2_ *A*_n_*/C*_a_ model as a new tool for *R*_d_ quantification in C_3_ crops. The findings enhance understanding of non-photosynthetic carbon flux dynamics and provide critical parameters for refining crop carbon cycling models to predict growth and yield under current and future atmospheric conditions.

## 1. Introduction

Leaf day respiratory rate (*R*_d_; see Table 1 for abbreviations and units)—defined as non-photorespiratory mitochondrial CO_2_ release in illuminated leaves—is a pivotal component of plant carbon metabolism. It directly modulates net photosynthetic carbon gain and overall plant fitness, distinguishing itself from dark respiration by occurring concurrently with photosynthesis and photorespiration (*R*_p_) [1–5]. This co-occurrence creates a long-standing challenge in plant ecophysiology: overlapping CO_2_ fluxes from photosynthetic fixation, photorespiratory release, and respiratory efflux complicate accurate *R*_d_ quantification using conventional gas-exchange techniques alone [6–10]. Accurate *R*_d_ estimation is critical for understanding plant carbon-use efficiency (CUE), as it accounts for 30–60% of gross photosynthetic carbon assimilation in terrestrial plants [11–15]. For C_3_ species, *R*_d_ interacts closely with *R*ₚ, a process that consumes 20–50% of photosynthetically fixed carbon under ambient conditions [1]. Together, *R*_d_ and *R*_p_ constitute the gross photorespiratory rate (*R*_C_ = *R*ₚ + *R*_d_), representing combined non-photosynthetic CO_2_ release that constrains net carbon assimilation [8,16–18]. Failure to disentangle *R*_d_ from *R*_p_ limits our ability to model plant responses to environmental changes (e.g., rising atmospheric CO_2_, global warming) [11,19].

**Table 1:** Definitions and units of key abbreviations used in this study.

Abbreviation	Definition	Units
*A*	Gross carbon assimilation rate	μmol m^–^ ^2^ s^–1^
*A*_n_	Net photosynthetic rate	μmol m^–2^ s^–1^
*A*_nmax_	Maximum net photosynthetic rate	μmol m^–2^ s^–1^
*A*_n_/*C*_i_ or *A*_n_/*C*_a_	CO_2_-response curve of photosynthesis	
*A*_n_/*I*	Light-response curve of photosynthesis	
*C*_a_	Ambient CO_2_ concentration	μmol mol^–1^
*C*_i_	Intercellular CO_2_ concentration	μmol mol^–1^
*C*_i,PTU_ or *C*_a,PTU_	Intercellular/atmospheric CO_2_ concentration transit point from ribulose-1,5-bisphosphate (RuBP)-limited to triose phosphate utilization (TPU)-limited photosynthesis	μmol mol^–1^
*R*_d_	Day respiratory rate	μmol m^–2^ s^–1^
*R*_p_	Photorespiratory rate	μmol m^–2^ s^–1^
*R*_C_	Gross photorespiratory rate	μmol m^–2^ s^–1^
*R*_C_/*C*_i_ or *R*_C_/*C*_a_	CO_2_-response curve of gross photorespiration	
*R*_C0_	Gross photorespiratory rate at *C*_a_ = 0 μmol mol^–1^	μmol m^–2^ s^–1^
*α*	Coefficient independent of *C*_i_ or *C*_a_	mol m^–2^ s^–1^
*β*	Rubisco oxygenation coefficient	mol μmol^–1^
*γ*	Rubisco carboxylation coefficient	mol μmol^–1^
*Γ*	CO_2_ compensation point in the presence of *R*_d_	μmol mol^–1^

Traditional *R*_d_ estimation methods—including the Kok method, Laisk method, and chlorophyll fluorescence (CF)-based Yin method—have inherent limitations [2,12,20–23]. The Kok method underestimates *R*_d_ by ignoring declines in photosystem II quantum efficiency with increasing irradiance; the Laisk method relies on low CO_2_ concentrations and fails for C_4_ species; and the Yin method struggles to account for CO_2_ reassimilation and *R*_d_-*R*ₚ metabolic interactions [3,5,9,20,21,24]. Notably, all these methods depend on low light or low CO_2_ conditions, with no prior studies exploring high CO_2_ (>1200 µmol mol^–1^) combined with low O_2_ (2%)—a regime we hypothesize can suppress *R*_p_ drastically: high CO_2_ competitively inhibits Rubisco oxygenation, while low O_2_ directly reduces *R*ₚ initiation, leaving *R*_C_ largely reflecting *R*_d_ alone.

*A*_n_/*C*_i_ (net photosynthesis *vs*. intercellular CO_2_) and *A*_n_/*C*_a_ (net photosynthesis *vs*. ambient CO_2_) curves have long characterized photosynthetic capacity, and their utility in resolving *R*_d_ and *R*ₚ has gained renewed attention [8,16]. These curves capture carboxylation, *R*ₚ, and *R*_d_ dynamics across CO_2_ gradients, providing a basis for mechanistic modeling of overlapping fluxes—without invasive isotope labeling or complex gas exchange-CF coupling [5,25]. However, the validity and robustness of this high-CO_2_/low-O_2_ approach for quantifying *R*_d_ in C_3_ plants had not been empirically tested. Our previous work focused on a single O_2_ environment and did not attempt to isolate *R*_d_ [26], which are the key issues that this study aims to address.

We hypothesize that high-CO_2_/low-O_2_ strongly suppresses photorespiration, so that *R*_c_ approximates true *R*_d_; this would be supported by *R*_d_ estimates from 2% O_2_ data being consistent with literature values and stable across O_2_ treatments. Using two complementary datasets—21% O_2_ (natural conditions) *A*_n_/*C*_a_ curves for four C_3_ species (*Trifolium pratense*, *Silphium perfoliatum*, *Lolium perenne*, *Triticum aestivum*) from Ye et al. [26], and our newly collected 2% O_2_ (photorespiration-suppressed) *A*_n_/*C*_a_ curves for the same species—this study aimed to address three core objectives: (1) Develop a robust analytical framework to extract *R*_d_-related parameters from *A*_n_/*C*ₐ curves under 21% and 2% O_2_; (2) Evaluate the consistency of 21% O_2_-derived *R*_d_ estimates with 2% O_2_ data under the assumption that *R*ₚ is minimized under low O_2_ (i.e., *R*_C_ ≈ true *R*_d_), and cross-reference these estimates with literature-derived *R*_d_ ranges for C_3_ crops [1,25,27]. It should be emphasized that this approach provides indirect support rather than independent validation of the *R*_C_ ≈ *R*_d_ assumption. (3) Analyze interspecific differences in *R*_d_ stability across O_2_ levels and link variability to species-specific photosynthetic traits.

## 2. Materials and methods

### 2.1. Model descriptive

Given that the precise measurement of *R*_d_ in plants remains a long-standing challenge [2,3,28] (for a comprehensive review, see Tcherkez et al. [8]), we developed a dedicated model to address this limitation. This model not only achieves accurate fitting of *A*_n_/*C*_i_ or *A*_n_/*C*_a_ curves but also enables the extraction of key measurable photosynthetic parameters. These parameters include: (1) the ambient CO_2_ concentration at the transition from RuBP-limited to TPU-limited photosynthesis (*C*_a,TPU_); (2) the maximum net photosynthetic rate (*A*_nmax_) corresponding to *C*_a,TPU_; and (3) the photorespiratory CO_2_ compensation point (*Γ*) in the presence of *R*_d_. Additionally, the model minimizes potential fitting discrepancies caused by variations in *R*_d_, addressing a critical shortcoming of conventional analytical approaches [26,29].

Equation 1 was developed by extending the non-asymptotic framework of Ye et al. [30] to incorporate both the carboxylation and oxygenation reactions of Rubisco. Under saturating or non-saturating irradiance levels, the model is expressed as follows:


$$\begin{array}{c} A_{\text{n}}=\alpha \frac{1-\beta C_{\text{a}}}{1+\gamma C_{\text{a}}}\left(C_{\text{a}}-\Gamma \right) \end{array}$$ (1)

where *A*_n_ (unit: mol m^–2^ s^–1^) is net photosynthetic rate, *C*_a_ (unit: mol mol^–1^) is the ambient CO_2_ concentration. *Γ* (unit: mol mol^–1^) is the CO_2_ compensation point in the presence of *R*_d_, representing the *C*_a_ at which *A*_n_ = 0. *α* (unit: mol m^–2^ s^–1^) is the coefficient independent of *C*_i_ or *C*_a_, reflecting the maximum carboxylation efficiency under CO_2_-limited conditions; *β* (unit: mol μmol^–1^) is the Rubisco oxygenation coefficient, which determines the rate at which *A*_n_ declines at high *C*_a_ due to photorespiratory carbon loss; *γ* (unit: mol mol^–1^) is the Rubisco carboxylation coefficient, which determines the curvature of the *A*_n_/*C*_a_ response and the onset of CO_2_ saturation.

Focusing on the parameters governing *A*_n_/*C*_a_ curve dynamics (*γ* and *β*), we found their values directly shape curve morphology and *A*_n_ responses to *C*_a_, with distinct patterns observed under controlled variable conditions (Fig 1). Specifically, when *γ* was held constant (Fig 1A, *γ* fixed at 0.0015 mol μmol^–1^), increasing *β* from 0.0000 to 0.00035 mol μmol^–1^ induced two distinct changes: (1) a more pronounced curve curvature (e.g., at *C*_a_ = 600 μmol mol^–1^, the curve slope for *β* = 0.00035 was 0.0071 mol m^–2^ s^–1^ per μmol mol^–1^, 40% steeper than that for *β* = 0.0000) and (2) a steeper decline in *A*_n_ as *C*_a_ exceeded 1000 μmol mol^–1^ (e.g., at *C*_a_ = 2000 μmol mol^–1^, *A*_n_ under *β* = 0.00035 was 4.71 μmol m^–2^ s^–1^, —only 18.6% of the *A*_n_ value observed at *β* = 0.0000). Conversely, when *β* was kept unchanged (Fig 1B, *β* fixed at 0.00005 mol μmol^–1^), higher *γ* (from 0.001 to 0.005 mol μmol^–1^) reduced curve curvature and slowed the rate of *A*_n_ increase with rising *C*_a_: at *C*_a_ = 600 μmol mol^–1^, the *A*_n_ increase rate for *γ* = 0.005 was 0.0055 mol m^–2^ s^–1^ per μmol mol^–1^, 29.7% slower than that for *γ* = 0.001; meanwhile, the *A*_n_ plateau (CO_2_ saturation point) appeared earlier (at ~1400 μmol mol^–1^ for *γ* = 0.005 *vs*. ~ 1800 μmol mol^–1^ for *γ* = 0.001).

**Fig 1:**
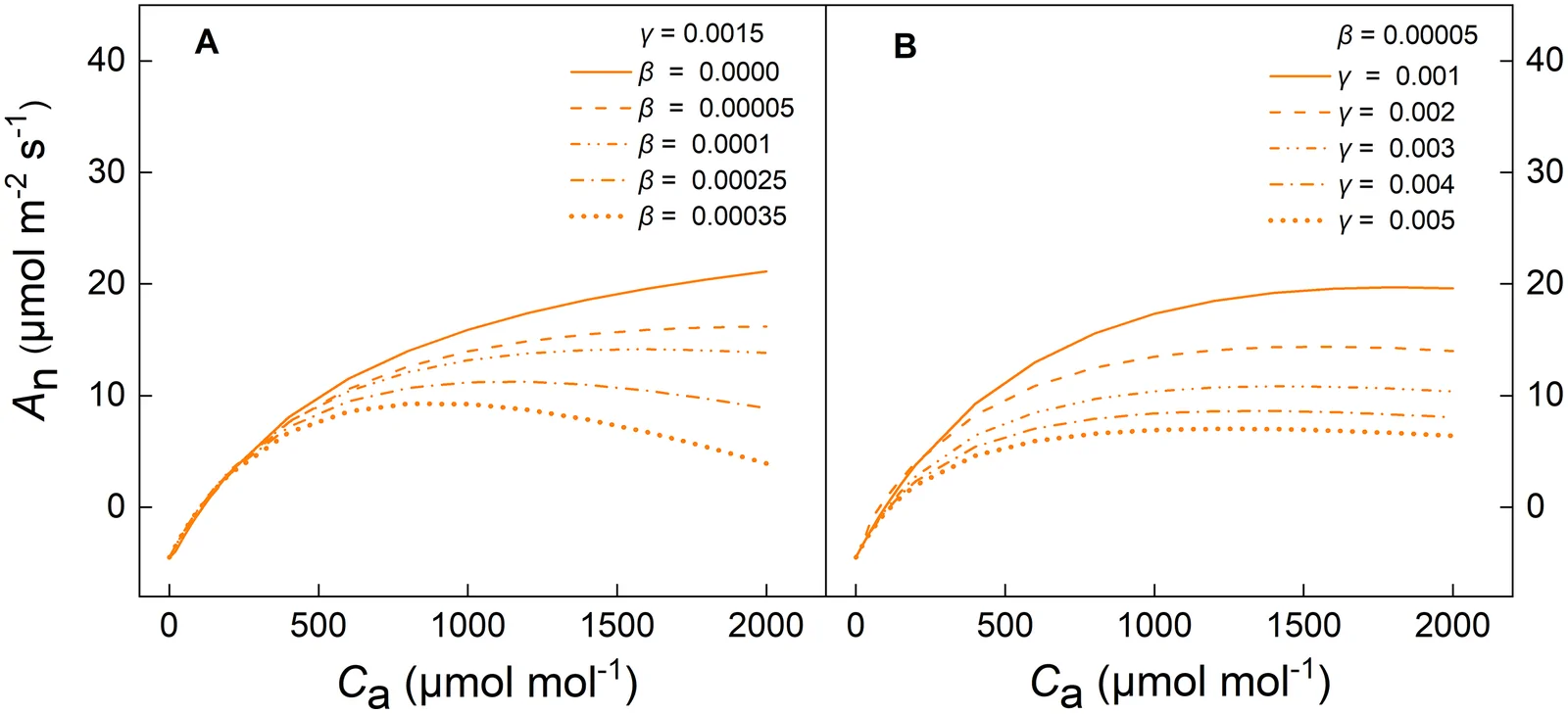
Effects of Rubisco carboxylation coefficient (*γ*) and oxygenation coefficient (*β*) in Eqn 1 on simulated *A*_n_*/C*_a_ curves under fixed parameters (*α* = 0.055 mol m^–2^ s^–1^, *Γ* = 103 μmol mol^–1^, *R*_C0_ = 5.4 μmol m^–2^ s^–1^). (A) *γ* fixed at 0.0015 mol μmol^–1^ with varying *β*; (B) *β* fixed at 0.00005 mol μmol^–1^ with varying *γ*.

These observations—consistent with the well-documented role of Rubisco-catalyzed reactions in modulating *A*_n_/*C*_a_ dynamics [16,31–34]—confirm that *β* is associated with Rubisco’s oxygenation reaction, while *γ* links to Rubisco’s carboxylation reaction.

Moreover, Eqn 1 can be rearranged as:


$$\begin{array}{c} A_{\text{n}}=\alpha \frac{1-\beta C_{\text{a}}}{1+\gamma C_{\text{a}}}C_{\text{a}}-\alpha \frac{1-\beta C_{\text{a}}}{1+\gamma C_{\text{a}}}\Gamma \end{array}$$ (2)

In Eqn 2, the first term denotes the gross carbon assimilation rate (*A*); however, the second term represents the gross photorespiration rate (*R*_C_), which incorporates *R*_p_ and *R*_d_ (*R*_C_ = *R*_p_ + *R*_d_) [17].

The first term in Eqn 2 can be expressed as:


$$\begin{array}{c} A=\alpha \frac{1-\beta C_{\text{a}}}{1+\gamma C_{\text{a}}}C_{\text{a}} \end{array}$$ (3)

Equation 3 demonstrates that *A* increases with rising *C*ₐ, until it reaches a maximum value (*A_max_*); subsequently, *A* decreases as *C*ₐ continues to increase. Thus, Eqn 3 is a non-asymptotic function.

And the *R*_C_ can be expressed as:


$$\begin{array}{c} R_{\text{C}}=\alpha \frac{1-\beta C_{\text{a}}}{1+\gamma C_{\text{a}}}\Gamma \end{array}$$ (4)

Equation 4 demonstrates that *R*_C_ (i.e., *R*_C_ = *R*_p_ + *R*_d_) decreases nonlinearly with rising *C*ₐ. Under 2% O_2_ and high *C*_a_, photorespiration is strongly suppressed and carboxylation dominates, so *R*_C_ ≈ *R*_d_. Under 2% O_2_ concentration and high *C*_a_ conditions, photorespiration is largely suppressed and carboxylation is high. In this case, *R*_C_ ≈ *R*_d_.

In this study, we initially fitted the *A*_n_/*C*ₐ curve with Eqn 1 to derive the parameters *α*, *β*, *γ*, and Γ_*_. These parameters were then substituted into Eqn 4, yielding a model that describes the response of *R*_C_ to *C*ₐ. This allows us to employ Eqn 4 to analyze *R*_C_ values at any given *C*ₐ. Specially, when *C*_a_ equals to 0 μmol mol^–1^, there is


$$\begin{array}{c} R_{\text{C0}}=\alpha \Gamma \end{array}$$ (5)

Combining Eqn 3 and Eqn 4, Eqn 2 can be expressed as:


$$\begin{array}{c} A_{\text{n}}=A-R_{\text{C}} \end{array}$$ (6a)

Compared with the FvCB model [16], i.e., *A*_n_ = *V*_c_−0.5*V*_o_−*R*_d_, Eqn 6a can be rewritten as follows:


$$\begin{array}{c} A_{\text{n}}=A-R_{\text{p}}-R_{\text{d}} \end{array}$$ (6b)

equation 6b can also be rearranged to yield the following expression:


$$\begin{array}{c} A=A_{\text{n}}+R_{\text{p}}+R_{\text{d}} \end{array}$$ (7)

Equation 7 explicitly represents the gross carbon assimilation rate.

Moreover, using Eqn 1, the *C*_a,TPU_ corresponding to the maximum net photosynthetic rate (*A*_nmax_) can be calculated by:


$$\begin{array}{c} C_{\text{a,TPU}}=\frac{\sqrt{\left(\beta +\gamma \right)\left(1\text{+}\gamma \Gamma \right)/\beta }-1}{\gamma } \end{array}$$ (8)

And the *A*_nmax_ corresponding to *C*_a,TPU_ can be obtained as:


$$\begin{array}{c} A_{\text{nmax}}=\alpha {\left[\frac{\sqrt{\beta +\gamma }-\sqrt{\beta \left(1\text{+}\gamma \Gamma \right)}}{\gamma }\right]}^{\text{2}} \end{array}$$ (9)

It should be noted that *C*_a_ can be substituted with intercellular CO_2_ concentration (*C*_i_) in Eqn 1. In this scenario, the values of *A*_nmax_ and *Γ* derived from Eqn 1 remain identical regardless of whether *C*_a_ or *C*_i_ is used. However, the parameters *C*_i,TPU_ (*C*_i_ corresponding to triose phosphate use limitation), *C*_a,TPU_, and *R*_C0_ (gross photorespiration rate when *C*_a_ approaches 0 μmol mol^–1^) will differ when *C*_a_ or *C*_i_ is employed as the input variable.

In this study, we quantitatively investigated the variation trends of *R*_C_ and *R*_d_ by fitting *A*_n_/*C*ₐ with Eqn 1. This approach was adopted because Eqn 1 enables the derivation of key photosynthetic parameters—including *A*_nmax_, *Γ*, and *R*_C0_—all of which are directly quantifiable from the fitted curves.

To evaluate whether Eqn 1 is a suitable mathematical tool for describing *A*_n_/*C*ₐ relationships in the tested C_3_ species, we further conducted an analysis of variance (ANOVA) to compare the parameter values derived from Eqn 1 with their corresponding observed values (e.g., measured *A*_n_ at specific *C*ₐ levels). A non-significant difference between calculated and observed values (*p* > 0.05) would indicate that Eqn 1 adequately describes the *A*_n_/*C*ₐ response for this experimental system. However, this comparison assesses model goodness-of-fit and does not constitute independent validation of the biological assumption that *R*_C_ ≈ *R*_d_ under 2% O_2_.

Moreover, Eqn 1 also has an alternative form that explicitly incorporates *R*_d_. Detailed information regarding this alternative form can be found in Ye et al*.* [26] and Kang et al*.* [29].

### 2.2. Materials and gas exchange measurement

In this study, we compiled and reanalyzed *A*_n_*/C*_a_ curves for a total of four C_3_ plant species, with data exclusively sourced from our own prior and ongoing research [26]. The species included in the analysis are *Trifolium pratense*, *Silphium perfoliatum*, *Lolium perenne*, and *Triticum aestivum*, for which *A*_n_*/C*_a_ curve data were initially reported under ambient oxygen concentration (21% O_2_) conditions [26]. For details regarding plant growth conditions, temperature, management procedures, and leaf selection criteria, please refer to the literature Ye et al. [26].

After simultaneously measuring the gas exchange and fluorescence data of four plant species under 21% O_2_, we further measured the gas exchange data and fluorescence data of these four plant species under 2% O_2_ under the same environmental conditions. The measurements of gas exchange and fluorescence data for the four plant species under different O_2_ concentrations were performed with three independent biological replicates (*n* = 3). Each biological replicate consisted of independently grown plants under identical conditions, and each *A*_n_*/C*_a_ curve was measured on a separate leaf. To establish a controlled low-oxygen environment, we fed a gas mixture containing 2% O_2_ (composed of 2% O_2_ and 98% N_2_, purchased from Ouyuan Gas Plant in Wenzhou City, Zhejiang Province) into a sealed plastic buffer bag through a pressure reducing valve. The buffer bag had a length of 1 meter and a width of 1.2 meters. A small amount of tap water was added to the buffer bag to mimic ambient humidity, preventing the leaves from losing water and drying out during the low-oxygen treatment. Subsequently, the buffer bag was connected to the Li-6400 portable photosynthesis measurement system via an inlet pipe, thereby ensuring a stable and suitable low-O_2_ atmosphere was maintained in the experimental environment to meet the requirements of the experimental conditions. This gas mixing device has been granted a Chinese National Utility Model Patent (Patent No.: ZL 2015 2 0174847.1). The remaining measurement procedures were consistent with those used under the 21% O_2_ concentration.

These *A*_n_*/C*_a_ curves were used to parameterize Eqn (1) within the Photosynthesis Model Simulation Software (PMSS; Zipiao Software Development Co., Ltd., China; available online at http://www.zipiao.tech/). This software enables the simultaneous extraction of key photosynthetic parameters—including *A*_nmax_, *C*_a,TPU_, *Γ*, and *R*_C0_—from a single *A*_n_*/C*_a_ curve. When fitting the *A*_n_/*C*ₐ curves using the PMSS software, and the number of iterations was set to 100, the convergence criterion was defined as a residual sum of squares (RSS) < 1 × 10^–6^. For the fitting parameters, the initial values of the *α*, *β*, *γ*, and *Γ* were set to 0.15, 0.0001, 0.001 and 40, respectively—with these initial values applied consistently under both at 21% O_2_ and 2% O_2_ conditions.

To ensure the accuracy of *R*_C0_ determination, a critical adjustment was made during data analysis: although the leaf chamber *C*ₐ was set to 0 μmol mol^–1^ during experiments, post-measurement data export revealed non-zero *C*_a_ values (1–9 μmol mol^–1^). Therefore, the minimum measured *C*ₐ for each sample was treated as 0 μmol mol^–1^ when calculating *R*_C0_, eliminating potential biases from residual CO_2_ in the leaf chamber.

### 2.3. Statistical analyses

Data are shown as mean ± SE (*n* = 3). Paired Student’s *t*-tests were used to compare estimated *A*_nmax_, *C*_a,TPU_, *Γ*, and *R*_C0_ (via Eqn 1) with corresponding to observed values of *A*_nmax_, *C*_a,TPU_, *Γ*, and *R*_C0_ for each species. One-way analysis of variance (ANOVA) was conducted to compare differences between the observed and calculated values of the same parameter (e.g., *A*_nmax_) for each individual species, with statistical significance defined at *p* < 0.05 using SPSS 18.5 (SPSS Inc., Chicago, IL). Figures were generated with Origin 2021 (OriginLab, Northampton) and Adobe Illustrator CS6 (Adobe Systems, California).

## 3. Results

### 3.1. Photosynthetic CO_2_-Response Curves (A_n_/C_a_) Under Different O_2_ Concentrations

Fig 2 presents the CO_2_-response curves of *A*_n_ against ambient CO_2_ concentration (*C*ₐ) (i.e., *A*_n_/*C*ₐ curves) for four C_3_ plant species, including *T. pratense* (A), *S. perfoliatum* (B), *L. perenne* (C), and *T. aestivum* (D), under two oxygen concentration treatments (21% and 2% O_2_) with saturating irradiance. The *A*_n_/*C*ₐ curves were fitted using the analytical model established in this study (Eqn 1), where orange dashed lines correspond to the fitting results under 21% O_2_ and light green solid lines represent the fitting results under 2% O_2_.

**Fig 2:**
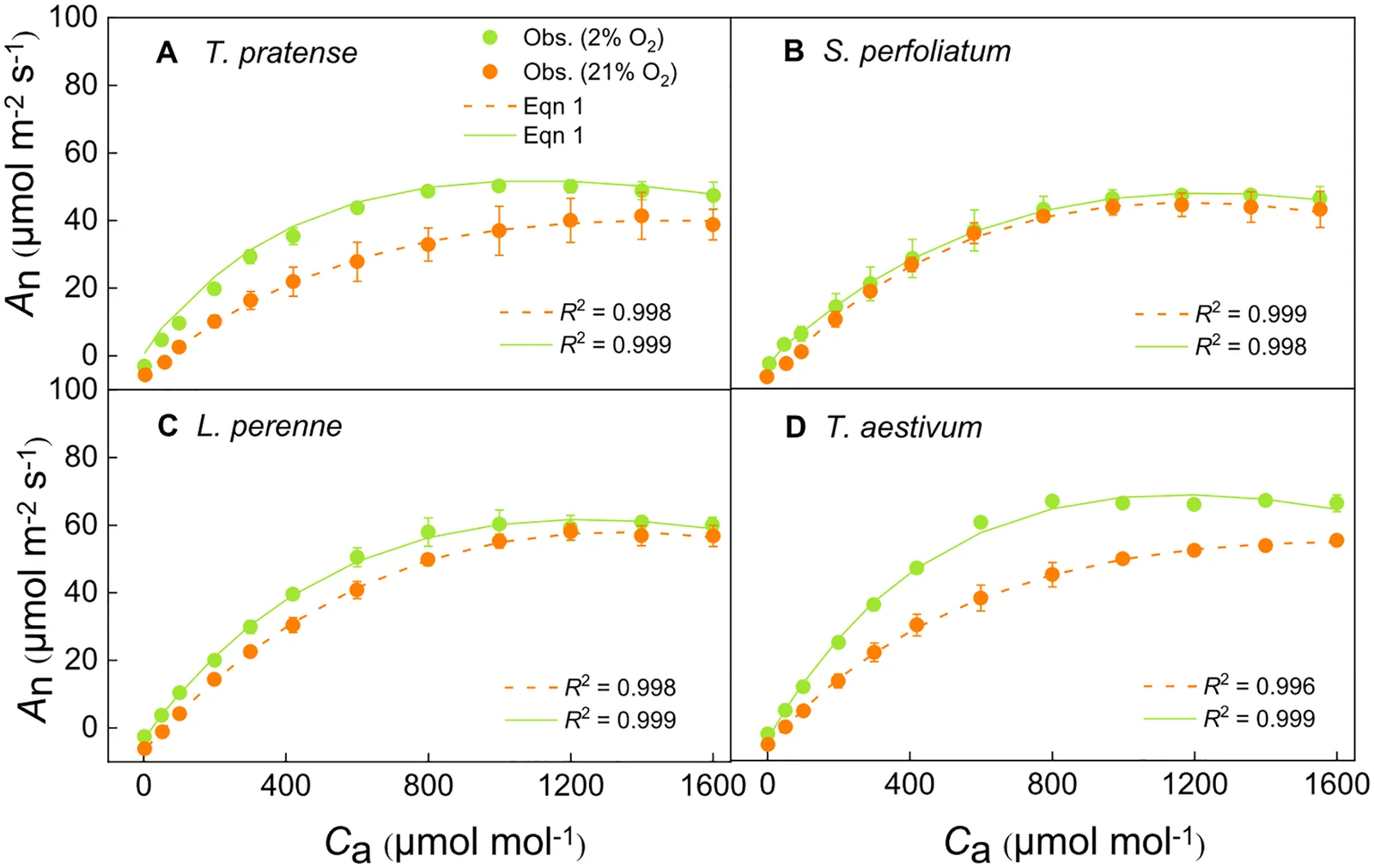
Net photosynthetic rate (*A*_n_) response to ambient CO_2_ concentration (*C*_a_) (*A*_n_*/C*_a_ curves) of four C_3_ plant species under 21% and 2% O_2_ concentrations with saturating irradiance. (A) *Trifolium pratense*, (B) *Silphium perfoliatum*, (C) *Lolium perenne*, (D) *Triticum aestivum*. Light green dots indicate observed data at 21% O_2_, and orange dots indicate observed data at 2% O_2_ (mean ± SE, *n* = 3); orange dashed lines and light green solid lines represent fittings of Eqn 1 for 21% and 2% O_2_, respectively.

Across all four species, the *A*_n_ values show a consistent trend of increasing with rising *C*ₐ at low to moderate CO_2_ concentrations, followed by a plateau at high *C*ₐ (≥1000 μmol mol^–1^), reflecting the typical CO_2_-saturation characteristic of C_3_ photosynthesis. Additionally, under the same *C*ₐ level, *A*_n_ values under 2% O_2_ are uniformly higher than those under 21% O_2_—consistent with the suppression of photorespiratory carbon loss under low O_2_ conditions. The close overlap between the observed data points (dots) and the model-fitted lines (dashed/solid), coupled with the consistently high coefficient of determination (*R*^2^ ≥ 0.996) across all species and O_2_ treatments.

### 3.2. Key photosynthetic parameters under 21% O_2_

In Table 2, the differences between the calculated (via Equations 1, 4, 8, and 9) and observed values of five key photosynthetic parameters—*C*_a,TPU_, *A*_nmax_, *Γ*, and *R*_C0_ (gross respiration rate at *C*_a_ ≈ 0 μmol mol^–1^)—for the four C_3_ species under 21% O_2_ were compared. Statistical analysis via paired *t*-test (*p* < 0.05) revealed no significant differences between calculated and observed values for any parameter. For *S. perfoliatum*, both *Γ* and *R*_C0_ were obtained by fitting *A*_n_*/C*ₐ curves (*C*_a_: 0–300 μmol mol^–1^) with Eqn 1.

**Table 2:** Comparison of calculated and observed key photosynthetic parameters for four C_3_ plant species under 21% O_2_ concentration.

Species	*C*_a,TPU_ (Eqn 8)	Observed *C*_a,TPU_	*A*_nmax_ (Eqn 9)	Observed *A*_nmax_	*Γ* (Eqn 1)	Observed *Γ*	*R*_C0_ (Eqn 4)	Observed *R*_C0_	*R* ^2^
*T. pratense*	1575.28 ± 140.11^a^	1399.40 ± 0.54^a^	40.51 ± 3.39^a^	41.36 ± 4.01^a^	67.81 ± 3.65^a^	72.53 ± 4.84^a^	5.70 ± 0.15^a^	5.65 ± 0.35^a^	0.999
*S. perfoliatum*	1228.37 ± 50.49^a^	1266.52 ± 176.03^a^	45.52 ± 1.84^a^	45.24 ± 1.95^a^	79.52 ± 4.27^a*^	84.92 ± 6.01^a^	6.41 ± 0.06^a*^	6.17 ± 0.25^a^	0.998
*L. perenne*	1408.42 ± 110.94^a^	1466.08 ± 132.77^a^	58.72 ± 0.23^a^	59.32 ± 0.72^a^	58.73 ± 1.08^a^	60.90 ± 0.46^a^	6.37 ± 0.51^a^	6.09 ± 0.28^a^	0.999
*T. aestivum*	1620.23 ± 129.73^a^	1599.08 ± 0.51^a^	54.95 ± 0.73^a^	55.09 ± 0.30^a^	50.94 ± 3.10^a^	51.14 ± 0.76^a^	5.71 ± 0.29^a^	5.31 ± 0.20^a^	0.999

**Note:** Calculated values were derived from Equations 1, 4, 8, and9; observed values are experimental measurements (mean ± SE, *n* = 3). Significance was tested by paired *t*-test (*p* < 0.05), with the same superscript “a” indicating no significant difference for the same parameter. Units: *A*_nmax_ and *R*_C0_ (μmol m^–2^ s^–1^); *C*_a,TPU_ and *Γ* (μmol mol^–1^). *Γ* and *R*_C0_ for *S. perfoliatum* were derived from fitting *A*_n_*/C*_a_ curves with *C*_a_ in the range of 0–300 μmol mol^–1^.

Among the species, *L. perenne* exhibited the highest calculated *A*_nmax_ (58.72 ± 0.23 μmol m^–2^ s^–1^), while *T. pratense* had the lowest calculated *A*_nmax_ (40.51 ± 3.39 μmol m^–2^ s^–1^). The calculated *Γ* values ranged from 50.94 ± 3.10 μmol mol^–1^ (*T. aestivum*) to 79.52 ± 4.27 μmol mol^–1^ (*S. perfoliatum*), and *R*_C0_ varied between 5.70 ± 0.15 μmol m^–2^ s^–1^ (*T. pratense* and *T. aestivum*) and 6.41 ± 0.06 μmol m^–2^ s^–1^ (*S. perfoliatum*). Additionally, *C*_a,TPU_ values were highest in *T. aestivum* (1620.23 ± 129.73 μmol mol^–1^) and lowest in *S. perfoliatum* (1228.37 ± 50.49 μmol mol^–1^), with no significant discrepancies between calculated and observed values for this parameter in any species (Table 2).

### 3.3. Key photosynthetic parameters Under 2% O_2_

Under 2% O_2_ (Table 3), the paired *t*-test (*p* < 0.05) again confirmed no significant differences between calculated and observed values for all tested photosynthetic parameters, consistent with the results under 21% O_2_. Likewise, the parameters *Γ* and *R*_C0_ for *T. aestivum* were obtained by fitting *A*_n_*/C*ₐ curves (*C*_a_: 0–300 μmol mol^–1^) with Eqn 1 under 2% O_2_.

**Table 3:** Comparison of calculated and observed key photosynthetic parameters for four C_3_ plant species under 2% O_2_ concentration.

Species	*C*_a,TPU_ (Eqn 8)	Observed *C*_a,TPU_	*A*_nmax_ (Eqn 9)	Observed *A*_nmax_	*Γ* (Eqn 1)	Observed *Γ*	*R*_C0_ (Eqn 4)	Observed *R*_C0_	*R* ^2^
*T. pratense*	1136.67 ± 51.75^a^	1266.54 ± 176.82^a^	50.79 ± 0.82^a^	50.77 ± 0.83^a^	24.86 ± 1.66^a^	24.74 ± 1.65^a^	3.26 ± 0.11^a^	3.11 ± 0.23^a^	0.998
*S. perfoliatum*	1280.33 ± 103.04^a^	1400.13 ± 115.71^a^	48.73 ± 0.31^a^	48.09 ± 0.68^a^	28.43 ± 4.58^a^	24.28 ± 3.36^a*^	2.57 ± 0.04^a^	2.19 ± 0.28^a^	0.999
*L. perenne*	1245.12 ± 28.85^a^	1333.27 ± 66.70^a^	61.72 ± 1.92^a^	60.89 ± 1.03^a^	25.22 ± 3.02^a^	24.74 ± 3.53^a^	3.04 ± 0.28^a^	2.52 ± 0.19^a^	0.998
*T. aestivum*	1164.57 ± 30.37^a^	1333.22 ± 175.64^a^	68.70 ± 0.49^a^	67.70 ± 0.69^a^	16.54 ± 0.85^a*^	16.32 ± 1.01^a^	2.29 ± 0.16^a*^	2.13 ± 0.19^a^	0.996

**Note:** Calculated values were derived from Equations 1, 4, 8, and 9; observed values are experimental measurements (mean ± SE, *n* = 3). Significance was tested by paired *t*-test (*p* < 0.05), with the same superscript “a” indicating no significant difference for the same parameter. Units: *A*_nmax_ and *R*_C0_ (μmol m^–2^ s^–1^); *C*_a,TPU_ and *Γ* (μmol mol^–1^). *Γ* and *R*_C0_ for *T. aestivum* were derived from fitting *A*_n_*/C*_a_ curves with *C*_a_ in the range of 0–300 μmol mol^–1^.

Notably, reducing O_2_ concentration from 21% to 2% induced consistent changes in specific parameters across species (Tables 2 and 3): (1) *Γ*: Significantly decreased in all species, with calculated values ranging from 16.54 ± 0.85 μmol mol^–1^ (*T. aestivum*) to 28.43 ± 4.58 μmol mol^–1^ (*S. perfoliatum*)—a reduction of ~50–67% compared to 21% O_2_. (2) *R*_C0_: Also declined, with calculated values spanning 2.13 ± 0.19 μmol m^–2^ s^–1^ (*T. aestivum*) to 3.11 ± 0.23 μmol m^–2^ s^–1^ (*T. pratense*)—a ~ 40–55% decrease relative to 21% O_2_. (3) *A*_nmax_: Tended to increase under 2% O_2_, with *T. aestivum* showing the highest calculated *A*_nmax_ (68.70 ± 0.49 μmol m^–2^ s^–1^) and *S. perfoliatum* the lowest (48.73 ± 0.31 μmol m^–2^ s^–1^); this represented a ~ 5–25% increase compared to 21% O_2_ for most species.

For *C*_a,TPU_, no consistent trend in response to O_2_ concentration was observed (Tables 2 and 3), with calculated values ranging from 1136.67 ± 51.75 μmol mol^–1^ (*T. pratense*) to 1280.33 ± 103.04 μmol mol^–1^ (*S. perfoliatum*) under 2% O_2_ (Table 3).

### 3.4. CO_2_-Response of Gross Photorespiratory Rate (R_C_/C_ₐ_) Across C_3_ Plant Species Under 21% and 2% O_2_

Fig 3 presents the CO_2_-response curves of the gross photorespiratory rate normalized to ambient CO_2_ concentration (*R*_C_/*C*ₐ) for four C_3_ plant species—*T. pratense* (A), *S. perfoliatum* (B), *L. perenne* (C), and *T. aestivum* (D)—under 21% and 2% O_2_ concentrations with saturating irradiance.

**Fig 3:**
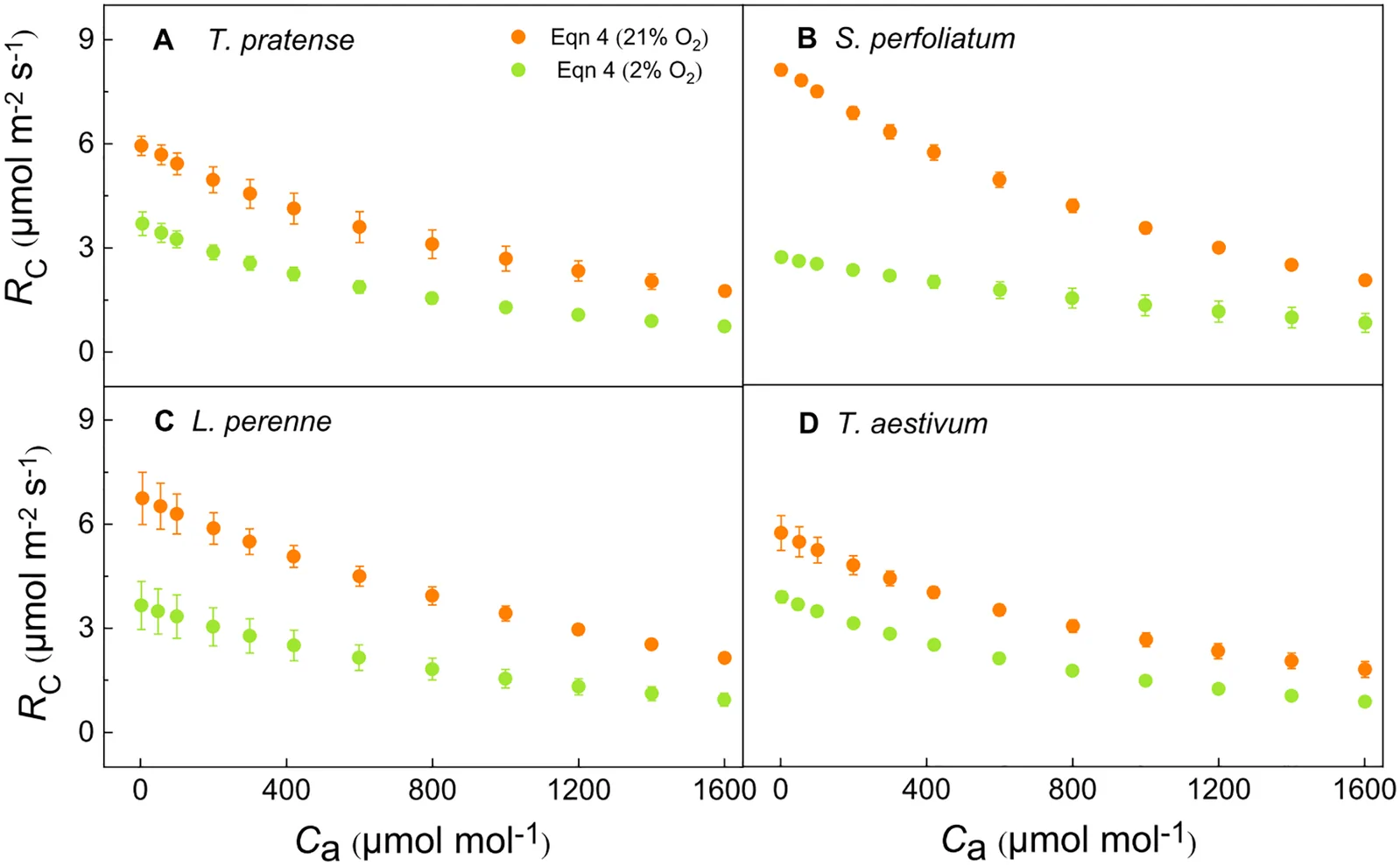
CO_2_-response curves of the gross photorespiratory rate normalized to ambient CO_2_ concentration (*R*_C_*/C*_a_ curves) of four C_3_ plant species under 21% and 2% O_2_ concentrations with saturating irradiance. (A) *Trifolium pratense*, (B) *Silphium perfoliatum*, (C) *Lolium perenne*, (D) *Triticum aestivum*. Orange dots indicate observed data at 21% O_2_, and light green dots indicate observed data at 2% O_2_ (mean ± SE, *n* = 3).

Across all C_3_ plant species, *R*_C_/*C*ₐ exhibited a consistent decreasing trend with increasing *C*ₐ, though the magnitude of this decline differed significantly between 21% and 2% O_2_ treatments. Under 21% O_2_, the initial *R*_C_ values (at low *C*_a_, ~ 0 μmol mol^–1^) were notably higher than those under 2% O_2_: for example, *L. perenne* (Fig 3C) showed an initial *R*_C_ of ~6.74 μmol m^–2^ s^–1^ at 21% O_2_, compared to ~3.65 μmol m^–2^ s^–1^ at 2% O_2_. As *C*ₐ increased to 1600 μmol mol^–1^, *R*_C_ plateaued in four C_3_ plant species, with 2% O_2_ treatments consistently yielding lower plateau values (ranging from ~0.74 to 0.94 μmol m^–2^ s^–1^) than 21% O_2_ (ranging from ~1.75 to 2.14 μmol m^–2^ s^–1^) (Fig 3). Interspecific differences were also evident: *L. perenne* (Fig 3C) maintained the highest *R*_C_ values across all *C*ₐ and O_2_ combinations, while *T. aestivum* (Fig 3D) had the lowest. For instance, at 600 μmol mol^–1^ *C*ₐ and 21% O_2_, *L. perenne* had an *R*_C_ of ~4.5 μmol m^–2^ s^–1^, whereas *T. aestivum* had a value of ~2.11 μmol m^–2^ s^–1^.

Table 4 presents the calculated *R*_C_ values via Eqn 4 for four C_3_ plant species (*T. pratense*, *S. perfoliatum*, *L. perenne*, *T. aestivum*) under 2% O_2_ concentration at three ambient CO_2_ concentrations (Cₐ: 1200, 1400, and 1600 μmol mol^–1^). The calculated *R*_C_ values under high-CO_2_/low-O_2_ (>1200 μmol mol^–1^ CO_2_ + 2% O_2_) conditions ranged from 0.74 to 1.12 μmol m^–2^ s^–1^. For *T. pratense* and *T. aestivum*, *R*_C_ values significantly decreased as *C*ₐ increased (e.g., *T. pratense*: 1.07 ± 0.09 μmol m^–2^ s^–1^ at 1200 μmol mol^–1^ *vs*. 0.74 ± 0.04 μmol m^–2^ s^–1^ at 1600 μmol mol^–1^, paired *t*-*t*est, *p* < 0.05), while no significant differences in *R*_C_ were observed across *C*ₐ levels for *S. perfoliatum* and *L. perenne* (*p* > 0.05). Among the species, *L. perenne* generally had the highest *R*_C_ at each Cₐ, and *T. pratense* the lowest at 1600 μmol mol^–1^ (Table 4).

**Table 4:** Calculated gross photorespiratory rate (*R*_C_) of four C_3_ plant species under 2% O_2_ concentration at three ambient CO_2_ concentrations (*C*_a_: 1200, 1400, and 1600 μmol mol^–1^).

Species	*T. pratense*	*S. perfoliatum*	*L. perenne*	*T. aestivum*
*R*_C_ at 1600 μmol mol^–1^	0.74 ± 0.04^b^	0.84 ± 0.17^a^	0.94 ± 0.11^a^	0.87 ± 0.02^c^
*R*_C_ at 1400 μmol mol^–1^	0.89 ± 0.07^ab^	0.99 ± 0.29^a^	1.12 ± 0.20^a^	1.04 ± 0.04^b^
*R*_C_ at 1200 μmol mol^–1^	1.07 ± 0.09^a^	1.16 ± 0.30^a^	1.32 ± 0.23^a^	1.24 ± 0.05^a^

**Note:** *R*_C_ values were calculated using Equation 4 (mean ± SE, *n* = 3). Significance was tested by paired *t*-test (*p* < 0.05): for the same species, different superscripts (“a,” “b,” “c”) indicate significant differences among *C*_a_ levels, while the same superscript indicates no significant difference. Unit: *R*_C_ (μmol m^–2^ s^–1^).

## 4. Discussion

### 4.1. Validity of the high-CO_2_/Low-O_2_ approach for estimating R_d_ in C_3_ plants

Our study demonstrates that combining high CO_2_ concentrations (>1200 µmol mol^–1^) with low O_2_ (2%) in *A*_n_/Cₐ curve measurements provides a robust experimental framework for estimating *R*_d_ in C_3_ species. Under these conditions, *R*_p_ is strongly suppressed: high CO_2_ competitively inhibits Rubisco oxygenation, while low O_2_ directly reduces substrate availability for this process [5,16,24,35]. Consequently, the gross photorespiratory rate (*R*_C_ = *R*ₚ + *R*_d_) measured under 2% O_2_ closely approximates *R*_d_—a conclusion supported by the significantly lower *R*_C0_ values (at 0 µmol mol^–1^ CO_2_) under 2% O_2_ compared to 21% O_2_ (Tables 2 and 3). This aligns with theoretical expectations that *R*ₚ is minimized under low-O_2_ conditions, enabling *R*_d_ isolation with minimal interference [2,3,5]. Importantly, the suppression of *R*ₚ under 2% O_2_ did not come at the cost of reduced photosynthetic capacity. In fact, *A*_max_ increased by approximately 5–25% under 2% O_2_ compared with 21% O_2_ across most species (Tables 2 and 3). This increase is consistent with the suppression of photorespiratory carbon loss, which allows a greater proportion of fixed carbon to be retained. This observation also supports that the low-O_2_ treatment did not induce hypoxic stress or impair the photosynthetic apparatus, thereby implying the use of 2% O_2_ as an appropriate condition for isolating *R*_d_ from *R*ₚ.

The reliability of our *R*_d_ estimates is supported by their consistency with previously reported ranges (0.52–1.75 µmol m^–2^ s^–1^) at 21% O_2_ and (0.74–2.89 µmol m^–2^ s^–1^) at 2% O_2_ for C_3_ crops under moderate temperatures [2]. However, it should be emphasized that this consistency provides indirect support rather than independent validation of the *R*_C_ ≈ *R*_d_ assumption, as no ground-truth measurements (e.g., ^13^C labeling) were conducted on the same leaves. Additionally, *R*_C_ values derived from 21% O_2_ curves (after accounting for *R*ₚ) match those from 2% O_2_ benchmarks, supporting the utility of our model (Eqn 1) for separating *R*_d_ from *R*ₚ without requiring invasive isotopic labeling or complex fluorescence coupling [5,35]. Nevertheless, this remains an indirect approach, and independent validation using ground-truth techniques is needed to confirm the *R*_C_ ≈ *R*_d_ assumption. For future studies, we recommend adding more low-*C*ₐ points (e.g., 30 and 80 µmol mol^–1^) to *A_n_*/Cₐ curves: these points will better constrain model fitting in the low-CO_2_ region, where respiration dominates net CO_2_ exchange, and thus improve the accuracy of *R*_C0_ and *Γ* estimates.

### 4.2. Interspecific variation in R_d_ and its relationship with photosynthetic traits

Notable interspecific differences in *R*_d_ emerged among the four C_3_ species: *L. perenne* exhibited the highest *R*_C_ values under both O_2_ conditions, while *T. aestivum* consistently showed the lowest (Fig 3; Table 4). These variations may stem from species-specific differences in respiratory substrate availability, mitochondrial density, or nitrogen allocation between photosynthetic and respiratory machinery [1,19]. The interspecific differences in *R*_d_ observed in this study (highest in *L. perenne*, lowest in *T. aestivum*) are consistent with published reports on species-specific respiratory traits. For example, Amthor [1] noted that species with higher carboxylation efficiency tend to have lower respiratory carbon loss, which aligns with our observation that *T. aestivum* (high carboxylation efficiency) had the lowest *R*_d_. Similarly, Tissue et al. [11] reported species-specific differences in leaf respiration across different canopy positions, supporting the view that respiratory traits are tightly coupled to photosynthetic characteristics. Furthermore, *R*_d_ remained stable across O_2_ levels (21% *vs*. 2%) in all species, indicating that *R*_d_ is largely independent of photorespiratory flux under short-term experimental conditions. This challenges the assumption that *R*_d_ is strongly inhibited by light or influenced by photorespiratory metabolites [3,7,9,27,36,37]. Instead, our results support the view that *R*_d_ is primarily driven by metabolic processes such as the oxidative pentose phosphate pathway and mitochondrial ATP production—processes that operate independently of *R*ₚ under steady-state conditions [5,17,38].

### 4.3. Implications for modeling carbon use efficiency and crop productivity

Accurate quantification of *R*_d_ is critical for improving predictions of carbon use efficiency (CUE) and crop productivity under future climate scenarios [1–3,19,25,37,39]. Our model (Eqn 1) offers a practical tool for integrating *R*_d_ into photosynthetic models, as it does not require additional fluorescence or isotopic data. By explicitly parameterizing *R*_C_ and *Γ*, the model enables more precise simulations of net carbon gain under varying CO_2_ and O_2_ conditions [26,29]. For example, the species-specific *R*_d_ values reported here can refine crop models (e.g., DSSAT, APSIM) that currently rely on generalized respiratory parameters.

To further improve estimates of key parameters like *A*_nmax_ and *C*_a,TPU_, we recommend adding more high-*C*ₐ points (e.g., 1300, 1400, 1500, and 1600 µmol mol^–1^) to future *A*_n_/Cₐ curves. These points will better capture the saturation phase of photosynthesis and reduce uncertainty in estimating the TPU-limited plateau [3,26,35,40–42]. Such methodological refinements, combined with the validated high-CO_2_/low-O_2_ approach, will enhance our ability to predict crop responses to elevated CO_2_ and optimize breeding strategies for improved carbon efficiency.

### 4.4. Comparative evaluation of methods for estimating R_d_

Our findings contribute to ongoing efforts to evaluate methods for estimating leaf *R*_d_. The high-CO_2_/low-O_2_ approach we used shares conceptual similarities with established indirect methods, particularly in disentangling photorespiration from mitochondrial respiration. A key point of comparison is the method proposed by Yin et al*.* [2], which combines gas exchange (GE) and chlorophyll fluorescence (CF) measurements.

Yin et al. [2] showed that their CF-based method produced *R*_d_ estimates that aligned well with those from the Laisk method (a widely used standard, despite its limitations) in C_3_ species. A major advantage of the CF method over the Kok method is its correction for the decline in PSII electron transport efficiency (*Φ*_2_) with increasing irradiance—even at low light levels [2]. In contrast, the Kok method assumes *Φ*_2_ remains constant, leading to a systematic ~20% underestimation of *R*_d_ compared to the CF method [2]. Our results—showing suppressed *R*_C_ under low O_2_—align with this principle: accounting for dynamic electron transport (or, in our case, minimizing *R*_p_) is critical for accurate respiratory flux estimation. For wheat (*T. aestivum*), the *R*_C_ calculated in this study (measured at 1600 μmol mol^–1^ CO_2_ and 2% O_2_, a condition where *R*_C_ approximates true *R*_d_) was 0.87 ± 0.02 μmol m^–2^ s^–1^. This *R*_d_ proxy was higher than both the chlorophyll fluorescence (CF)-based *R*_d_ estimate at 2% O_2_ (0.69 ± 0.09 μmol m^–2^ s^–1^) and the Kok method-derived *R*_d_ estimate at 21% O_2_ (0.631 ± 0.151 μmol m^–2^ s^–1^). In contrast, it was lower than the Laisk method-derived *R*_d_ estimate at 21% O_2_ (0.97 ± 0.52 μmol m^–2^ s^–1^) and the CF-based *R*_d_ estimate at 21% O_2_ (1.043 ± 0.14 μmol m^–2^ s^–1^)—a pattern consistent with previously reported inter-method variability in respiratory flux quantification [2].

Notably, the Laisk method is inapplicable to C_4_ plants due to their CO_2_-concentrating mechanism, which invalidates the method’s underlying assumptions and can yield physiologically impossible negative *R*_d_ values (Fig 1B) [2]. In contrast, both the Kok and CF methods work for C_3_ and C_4_ species. While our study focused on C_3_ plants, the high-CO_2_/low-O_2_ framework in this study (like the CF method) does not depend on C_3_-specific metabolism, suggesting it could be adapted for C_4_ species in future research.

A practical consideration is that the CF method typically requires small leaf chambers (e.g., 2 cm^2^) for reliable fluorescence measurements, which increases the noise-to-signal ratio for gas exchange data compared to the larger chambers usable with the Kok or Laisk methods [2,13,20,21,23,43]. Our setup—without CF—allowed standard chamber sizes, highlighting a trade-off between parameter accuracy and measurement practicality. However, the development of area-imaging fluorescence systems may soon resolve this limitation of the CF method.

In summary, insights from Yin et al. [2] underscore that our *A*_n_/Cₐ curve approach (under *R*ₚ-suppressing conditions) is a viable, robust alternative for estimating *R*_d_. It avoids the need for complex fluorescence instrumentation while effectively isolating the respiratory component, yielding results theoretically aligned with the more advanced CF-based technique.

### 4.5. Limitations and field application of the high-CO_2_/low-O_2_ approach

It should be acknowledged that a key limitation of this study is that the *R*_C_ ≈ *R*_d_ assumption under 2% O_2_ was not independently validated using ground-truth measurements (e.g., ^13^C labeling or isotope ratio mass spectrometry) on the same leaves. In addition, the sample size (*n* = 3) represents a further limitation, and increasing the number of biological replicates in future studies would help further validate the method and improve statistical power. Although this assumption is supported by theoretical considerations (i.e., suppression of Rubisco oxygenation under low O_2_), by the substantial decrease in *Γ* (50–67%) and *R*_CO_ (40–55%) under 2% O_2_, and by the consistency of our *R*_d_ estimates with literature ranges, it remains a theoretical inference supported by indirect evidence rather than direct proof. Future studies should validate this approach by combining the high-CO_2_/low-O_2_ *A*_n_/*C*ₐ method with independent isotope-based measurements of *R*_d_ on the same leaves.

Although our measurements were conducted under controlled conditions, the core principle—suppressing photorespiration through high CO_2_ and low O_2_—can be adapted for field use. The portable buffer-bag system (Chinese National Utility Model Patent ZL 2015 2 0174847.1) enables stable gas delivery with portable gas exchange systems and requires only a CO_2_ source and a low-O_2_ gas mixture. However, field environmental fluctuations (e.g., temperature, humidity) may affect measurement stability and should be carefully monitored. This method can be applied to screen crop genotypes for respiratory traits under field-relevant conditions and to provide species-specific *R*_d_ parameters for crop models (e.g., DSSAT, APSIM). Future work should optimize the protocol for ambient CO_2_, develop automated gas mixing systems, and validate the method under field-relevant stresses (e.g., drought, heat).

## 5. Conclusion

This study systematically supports the efficacy of the high-CO_2_ (>1200 µmol mol^–1^)/low-O_2_ (2%) *A*_n_/*C*ₐ curve approach for the quantification of *R*_d_ in C_3_ species. A core mechanistic advantage of this framework lies in its ability to suppress *R*_p_: elevated CO_2_ competitively inhibits Rubisco oxygenation, while reduced O_2_ directly limits *R*ₚ initiation, thereby rendering the gross photorespiratory rate (*R*_C_ = *R*ₚ + *R*_d_) under 2% O_2_ a reliable proxy for true *R*_d_. The *R*_d_ estimates derived herein (0.74–0.94 µmol m^–2^ s^–1^) are consistent with established literature ranges for C_3_ species [1,2,27], further confirming the method’s reliability.

The dedicated analytical model (Eqn 1) developed in this study exhibits high fitting accuracy (*R*^2^ ≥ 0.996) across all tested C_3_ species, with paired *t*-tests (*p* > 0.05) revealing no significant discrepancies between calculated and observed photosynthetic parameters (e.g., *A*_nmax_, *Γ*, and *R*_C0_). This confirms the model’s capacity to disentangle *R*_d_ from overlapping *R*ₚ fluxes—a key limitation of conventional approaches. Interspecific variation in *R*_d_ is observed, with the highest value in *L. perenne* and the lowest in *T. aestivum*. This variation is tightly coupled to species-specific photosynthetic traits, particularly carboxylation efficiency. These findings suggest a potential trade-off between photosynthetic carbon fixation capacity and respiratory carbon loss. Critically, *R*_d_ remains stable across O_2_ concentrations (21% *vs*. 2%), which supports the hypothesis that *R*_d_ is driven by intrinsic metabolic processes (e.g., mitochondrial ATP synthesis, oxidative pentose phosphate pathway) rather than short-term fluctuations in photorespiratory flux [5,38].

Collectively, the species-specific *R*_d_ parameters generated in this study address the current reliance on generalized respiratory coefficients in crop carbon cycling models (e.g., DSSAT, APSIM), thereby enhancing the precision of predictions for CUE and crop productivity under future atmospheric CO_2_ enrichment scenarios.

Future research directions and priorities are as follows:

1. Broaden Applicability Across Species and Ecosystems: Validate the high-CO_2_/low-O_2_ framework in C_4_ crops (e.g., *Zea mays*, *Sorghum bicolor*) to address traditional method limitations (e.g., invalid Laisk method for C_4_ species) [2], and extend it to wild C_3_ species and diverse ecosystems (e.g., grasslands, forests) to enhance global carbon cycle modeling relevance.
2. Refine the Method for Real-World Field Conditions: Optimize the protocol for ambient CO_2_ (~420 µmol mol^–1^) using intermediate CO_2_ gradients—consistent with the field measurement context of this study—and validate it with gold-standard techniques (e.g., ^13^C labeling, time-resolved fluorescence). The portable buffer-bag system used here provides a practical basis for field deployment; future work should focus on developing automated gas mixing systems and validating the method under field-relevant abiotic stresses (e.g., drought, heat) to enhance its adaptability to practical agronomic scenarios.
3. Strengthen Mechanistic Insight and Standardization: Link interspecific *R*_d_ variability to physiological traits (mitochondrial density, substrate pools) and omics data to uncover regulatory pathways [1,19]. Establish a standardized protocol (gas exchange parameters, data processing) and conduct inter-laboratory tests to ensure reproducibility.

## Supporting information

S1 File This document contains raw data of Figs 1–3. (XLSX)
