# Supplementary material for: Disentangling leaf day respiration from photorespiration in C3 leaves: A single-curve An/Ca approach validated under photorespiration-suppressed conditions
Source: PLoS One. 2026 Oct 23;21(10):e0361238. doi: 10.1371/journal.pone.0361238 (PMC13693880; doi:10.1371/journal.pone.0361238)
Supplement: Supplementary file 1 [file pone.0361238.s002.docx]

**Disentangling Leaf Day Respiration from Photorespiration in C3 Leaves: A Single-Curve An/Ca Approach Validated under Photorespiration-Suppressed Conditions**

In this manuscript leaf day respiration as an important component of plant carbon metabolism that directly modulates net photosynthetic carbon gain and plant fitness, has long been challenging to accurately quantify due to overlapping CO₂ fluxes with photosynthesis and photorespiration (Rp). An/Ca curve data of four C3 species (Trifolium pratense, Silphium perfoliatum, Lolium perenne, Triticum aestivum) under ambient O2 (21% O2) from Ye et al. (2025) and newly collected data under low O2 (2% O2) for the same species was integrated here. A dedicated model (Eqn 1) was developed to extract key parameters [e.g., the maximum net photosynthetic rate (Anmax), CO2 compensation point (Γ) in the presence of Rd, photorespiratory rate at Ca = 0 μmol mol–1 (RC0)] from An/Ca curves, with ANOVA confirming no significant differences between calculated and observed parameter values (p > 0.05). Under 2% O2, Rp was strongly suppressed, making the gross photorespiratory rate (RC = Rp + Rd) a reliable proxy for approximate Rd. The statistical data was presented well and the manuscript has been written in clear and understandable language.
