# Supplementary material for: Disentangling leaf day respiration from photorespiration in C3 leaves: A single-curve An/Ca approach validated under photorespiration-suppressed conditions
Source: PLoS One. 2026 Oct 23;21(10):e0361238. doi: 10.1371/journal.pone.0361238 (PMC13693880; doi:10.1371/journal.pone.0361238)
Supplement: Peer review history

# Peer review: Decision Letter 0

**DOI:** 10.1371/journal.pone.0361238.r001

**Authors:** Thomas Roach

19 Aug 2026

Dear Dr. Wang,

Please submit your revised manuscript by Oct 18 2026 11:59PM. If you will need more time than this to complete your revisions, please reply to this message or contact the journal office at plosone@plos.org. When you're ready to submit your revision, log on to https://www.editorialmanager.com/pone/ and select the 'Submissions Needing Revision' folder to locate your manuscript file.

- A letter that responds to each point raised by the academic editor and reviewer(s). You should upload this letter as a separate file labeled 'Response to Reviewers'.
- A marked-up copy of your manuscript that highlights changes made to the original version. You should upload this as a separate file labeled 'Revised Manuscript with Track Changes'.
- An unmarked version of your revised paper without tracked changes. You should upload this as a separate file labeled 'Manuscript'.

If you would like to make changes to your financial disclosure, please include your updated statement in your cover letter. Guidelines for resubmitting your figure files are available below the reviewer comments at the end of this letter.

If applicable, we recommend that you deposit your laboratory protocols in protocols.io to enhance the reproducibility of your results. Protocols.io assigns your protocol its own identifier (DOI) so that it can be cited independently in the future. For instructions see: https://journals.plos.org/plosone/s/submission-guidelines#loc-laboratory-protocols. Additionally, PLOS One offers an option for publishing peer-reviewed Lab Protocol articles, which describe protocols hosted on protocols.io. Read more information on sharing protocols at https://plos.org/protocols?utm_medium=editorial-email&utm_source=authorletters&utm_campaign=protocols.

As the corresponding author, your ORCID iD is verified in the submission system and will appear in the published article. PLOS supports the use of ORCID, and we encourage all coauthors to register for an ORCID iD and use it as well. Please encourage your coauthors to verify their ORCID iD within the submission system before final acceptance, as unverified ORCID iDs will not appear in the published article. *Only*  the individual author can complete the verification step; PLOS staff *cannot*  verify ORCID iDs on behalf of authors.

We look forward to receiving your revised manuscript.

Kind regards,

Thomas Roach

Academic Editor

PLOS One

Journal Requirements:

When submitting your revision, we need you to address these additional requirements.

1. Please ensure that your manuscript meets PLOS ONE's style requirements, including those for file naming. The PLOS ONE style templates can be found at

https://journals.plos.org/plosone/s/file?id=wjVg/PLOSOne_formatting_sample_main_body.pdf and

https://journals.plos.org/plosone/s/file?id=ba62/PLOSOne_formatting_sample_title_authors_affiliations.pdf

2. Please note that PLOS One has specific guidelines on code sharing for submissions in which author-generated code underpins the findings in the manuscript. In these cases, we expect all author-generated code to be made available without restrictions upon publication of the work. Please review our guidelines at https://journals.plos.org/plosone/s/materials-and-software-sharing#loc-sharing-code and ensure that your code is shared in a way that follows best practice and facilitates reproducibility and reuse.

3. Thank you for stating the following in the Acknowledgments Section of your manuscript:

"This research was funded by the Natural Science Foundation of China (No. 32260063 and 32060428), the Natural Science Foundation of Jiangxi Province (Grant No.20224BAB205020), and the Natural Science Foundation of Ji'an City Science and Technology Plan, Jiangxi Province (Grant No. 20255- 071622)."

We note that you have provided additional information within the Acknowledgements Section that is not currently declared in your Funding Statement. Please note that funding information should not appear in the Acknowledgments section or other areas of your manuscript. We will only publish funding information present in the Funding Statement section of the online submission form.

Please remove any funding-related text from the manuscript and let us know how you would like to update your Funding Statement. Currently, your Funding Statement reads as follows:

"is research was funded by the Natural Science Foundation of China (No. 32260063 and 32060428), the Natural Science Foundation of Jiangxi Province (Grant No.20224BAB205020), and the Natural Science Foundation of Ji'an City Science and Technology Plan, Jiangxi Province (Grant No. 20255-071622)."

Please include your amended statements within your cover letter; we will change the online submission form on your behalf.

4.  Thank you for stating the following financial disclosure:

"This research was funded by the Natural Science Foundation of China (No. 32260063 and 32060428), the Natural Science Foundation of Jiangxi Province (Grant No.20224BAB205020), and the Natural Science Foundation of Ji'an City Science and Technology Plan, Jiangxi Province (Grant No. 20255-071622)."

Please state what role the funders took in the study.  If the funders had no role, please state: "The funders had no role in study design, data collection and analysis, decision to publish, or preparation of the manuscript."

If this statement is not correct you must amend it as needed.

Please include this amended Role of Funder statement in your cover letter; we will change the online submission form on your behalf.

5. Please provide a complete Data Availability Statement in the submission form, ensuring you include all necessary access information or a reason for why you are unable to make your data freely accessible. If your research concerns only data provided within your submission, please write "All data are in the manuscript and/or supporting information files" as your Data Availability Statement.

6. If the reviewer comments include a recommendation to cite specific previously published works, please review and evaluate these publications to determine whether they are relevant and should be cited. There is no requirement to cite these works unless the editor has indicated otherwise.

Additional Editor Comments:

[Note: HTML markup is below. Please do not edit.]

Reviewers' comments:

Reviewer's Responses to Questions

**Comments to the Author**

1. Is the manuscript technically sound, and do the data support the conclusions?

Reviewer #1: Yes

Reviewer #2: Yes

Reviewer #3: Yes

Reviewer #4: Yes

**********

2. Has the statistical analysis been performed appropriately and rigorously?

Reviewer #1: I Don't Know

Reviewer #2: Yes

Reviewer #3: No

Reviewer #4: Yes

**********

3. Have the authors made all data underlying the findings in their manuscript fully available?

The PLOS Data policy

Reviewer #1: Yes

Reviewer #2: Yes

Reviewer #3: Yes

Reviewer #4: Yes

**********

4. Is the manuscript presented in an intelligible fashion and written in standard English?

Reviewer #1: Yes

Reviewer #2: Yes

Reviewer #3: Yes

Reviewer #4: Yes

**********

Reviewer #1: This manuscript seems to be an important contribution to plant physiology. All the sections are rigorously articulated. However, there are some areas which needs to be improved before the paper is being published. Let them breakdown in the following sections.

Abstract:

• If possible, quantify the degree of suppression of photorespiration under 2% O2.

• Reduce use of strong statements such as “validated”, instead use “suggested”, “supported”.

• Clarify haw equation 1 was developed.

Introduction

• This section should be concise and to the point. Shorten it and remove repetition of some descriptions.

• Justify clearly, why the new method is needed.

• The knowledge gap which is addressed by the proposed model may be identified.

• The hypothesis put forward may be written more clearly and explicitly.

Material and methods

• Why 2% O2 is selected as suppressed condition of photorespiration.

• Any evidence of minimization of photosynthesis under these conditions.

• Equation 1, if explained would be better.

• Are these parameters physiologically significant?

• Statistically speaking, increase number of replications.

Results

• Don’t interpret biological data in this section.

• Compare oxygen treatment and species.

Discussion

• Compare your results with published work.

• Discuss limitations, if any.

• Discuss how it can be applied in field.

Figures and Graphs

• Improve figures for readability.

Conclusion

• Give future perspective for improvement.

• Highlight study limitations.

References

• Format according to journal style.

Overall recommendations

• Thoroughly check for English grammar correction and typos.

• Recheck the abbreviations.

Reviewer #2: The statistical data has been given in detail and written in an intelligible fashion. The manuscript is sound and the authors have made the findings available. The statistical data has been given in detail for the readers to understand the leaf day respiration

Reviewer #3: The manuscript addresses an important and difficult problem in leaf gas-exchange physiology, with the four-species, two O2 - level dataset is a quite reasonable empirical foundation but in its current form the manuscript has three major weaknesses that does not support the central claim that this is a “validated” method for isolating Rd from Rp.

1. The validation logic is circular i.e. Eqn 1 reproducing its own input data, is repeatedly presented as if it validates the biological assumption that RC approximates Rd under the chosen conditions. No independent ground - reality measurement on the same leaves is presented.

2. The results are internally inconsistent i.e. unit assignments in the model are not dimensionally coherent, Figures 2 and 3 display units that contradict the surrounding text and tables, and three different Rd ranges are reported for what should be a single caption number.

3. Main experimental controls are unverified as the actual O2 concentration delivered by the buffer - bag system over the course of a measurement is not reported, despite this being the essential operation of entire study.

Reviewer #4: The study on very important and applied aspect of plant physiology has been performed with standard protocols and it sounds technically excellent. The conclusions drwan are very relevent to data presented in text,tables and figures. It has been supported with future prospects of the study...The data generated has been rigorously and statistically analysed using latest models and softwares. And the data is visible in tabulted as well as figures form and no part is hiddden. The flow and sequence of the whole manuscript is very meaningful and self explanatory. The subheadinds explaining different aspects and parametres related to photosynthesis, photorespiration and day respiration are impressive in results as well as in discussion section. The sequence, placement and explanations of mathematical equations is excellent. Specially the explanations of results of application of new model for separating day resiration from photorespiration is very impressive. All this has been narrated in standard English language without any difficulty to understand by non english persons as well....Overall this MS has revealed understanding of future Co2 concentrations and its impact calculation for C3 species which are prone to photorespiration.

**********

what does this mean?). If published, this will include your full peer review and any attached files.

If you choose “no”, your identity will remain anonymous but your review may still be made public.

**Do you want your identity to be public for this peer review?**  For information about this choice, including consent withdrawal, please see our Privacy Policy

Reviewer #1: **Yes:**  Syed Hassan Ali Shah

Reviewer #2: **Yes:**  Tanushree Agarwal

Reviewer #3: No

Reviewer #4: No

**********

[NOTE: If reviewer comments were submitted as an attachment file, they will be attached to this email and accessible via the submission site. Please log into your account, locate the manuscript record, and check for the action link "View Attachments". If this link does not appear, there are no attachment files.]

To ensure your figures meet our technical requirements, please review our figure guidelines: https://journals.plos.org/plosone/s/figures

You may also use PLOS’s free figure tool, NAAS, to help you prepare publication quality figures: https://journals.plos.org/plosone/s/figures#loc-tools-for-figure-preparation.

NAAS will assess whether your figures meet our technical requirements by comparing each figure against our figure specifications.

Attachment Submitted filename: plosone.docx

# Author comment: Author response to Decision Letter 1

**DOI:** 10.1371/journal.pone.0361238.r002

23 Sep 2026

Dear editor：

Thank you very much for your letter with the reviewers’ comments on our manuscript "Disentangling Leaf Day Respiration from Photorespiration in C3 Leaves: A Single-Curve An/Ca Approach Validated under Photorespiration-Suppressed Conditions (Manuscript ID: PONE-D-26-26924)". These suggestions and comments have an important role in improving our manuscript, thanks. Now, we have carefully revised the original version (Manuscript ID: PONE-D-26-26924) according to the referee’s comments and suggestions and have made corrections which we hope meet with approval. The modification and correction are highlighted in red color in the revised manuscript.

Best regards,

Sincerely yours,

PhD & Associate Professor Fu-Biao Wang

-------------------------------------------------------------------------------------------------------

The following is a point-to-point response to your suggestions and comments.

Reviewer #1:

This manuscript seems to be an important contribution to plant physiology. All the sections are rigorously articulated. However, there are some areas which needs to be improved before the paper is being published. Let them breakdown in the following sections.

Response: We sincerely thank you for the positive assessment and the constructive comments. We have carefully addressed each point in the revised manuscript. Below we provide our point-by-point responses.

Abstract:

Q1• If possible, quantify the degree of suppression of photorespiration under 2% O2.

Response: We thank you for this valuable suggestion. In the revised Abstract, we have added quantitative information on the degree of photorespiration suppression. Specifically, we now state that under 2% O2, the CO2 compensation point (Γ) decreased by approximately 50–67% and the gross photorespiratory rate at Ca = 0 (RCO) decreased by approximately 40–55% compared with 21% O2. These quantitative changes provide direct evidence that photorespiration was substantially suppressed under low O2.

Q2• Reduce use of strong statements such as “validated”, instead use “suggested”, “supported”.

Response: This comment has been addressed in the revised manuscript. We have replaced "validated" with "suggested" or "supported" in multiple places throughout the manuscript. Thank you for your valuable suggestion.

Q3• Clarify haw equation 1 was developed.

Response: Thank you for your comments and suggestions. In the revised manuscript, we have supplemented the derivation of Equation 1 and the meaning of each parameter in the Materials and Methods section. Specific revisions can be found in Lines 95–96 and 100–105 of the revised manuscript.

Introduction

Q4• This section should be concise and to the point. Shorten it and remove repetition of some descriptions.

Response: Thank you for your constructive suggestions. We agree that the Introduction was overly long and contained repetitive descriptions. In the revised manuscript, we have substantially shortened the Introduction and merged several paragraphs to enhance the logical flow and readability of the paper. Specific revisions include: (1) We have consolidated the descriptions of traditional Rd estimation methods (Kok, Laisk, and Yin methods) into a single, more concise paragraph, removing redundant explanations of each method's limitations. (2) We have removed repeated statements about the importance of Rd quantification (e.g., its role in carbon-use efficiency and crop productivity), which overlapped with other paragraphs. (3) We have eliminated redundant descriptions of the overlapping CO2 fluxes from photosynthesis, photorespiration, and respiration. (4) We have also merged the last paragraph of the original manuscript into the third and fourth paragraphs of the revised manuscript. We believe that the logical flow and readability of the revised Introduction have been significantly improved. Specific revisions can be found in Lines 55, 60–61, 64–65, 70–75, and 79–84 of the revised manuscript.

Q5• Justify clearly, why the new method is needed.

Response: Thank you for your important suggestion. In the Introduction of the revised manuscript, we have provided a clear and explicit justification for the new method. First, existing methods have obvious limitations: all traditional methods (Kok, Laisk, and CF-based Yin methods) rely on low-light or low-CO2 conditions and have inherent limitations: the Kok method systematically underestimates Rd; the Laisk method is inapplicable to C4 species; and the Yin method requires complex fluorescence instrumentation and struggles to account for CO2 reassimilation. Second, to date, no study has explored the combination of high CO2 (>1200 μmol mol⁻¹) with low O2 (2%) as a means to suppress photorespiration and isolate Rd. There is an urgent need for a simple, robust, and fluorescence-independent approach for quantifying Rd in C3 crops. Specific revisions can be found in Lines 57–65 and 70–75 of the revised manuscript.

Q6• The knowledge gap which is addressed by the proposed model may be identified.

Response: We thank the reviewer for this suggestion. In the revised Introduction, we have explicitly identified the knowledge gap addressed by our proposed model. Specifically, we now state: "However, the validity and robustness of this high-CO2/low-O2 approach for quantifying Rd in C3 plants had not been empirically tested. Previous work by Ye et al. [26] focused on a single O2 environment and did not attempt to isolate Rd, leaving a clear knowledge gap that our study addresses." Specific revisions can be found in Lines 70–72.

Q7• The hypothesis put forward may be written more clearly and explicitly.

Response: This suggestion has been addressed in the revised manuscript. In the Introduction section of the revised manuscript, we have added a clear and explicit hypothesis statement: "We hypothesize that high-CO2/low-O2 strongly suppresses photorespiration, so that RC approximates true Rd; this would be supported by Rd estimates from 2% O2 data being consistent with literature values and stable across O2 treatments. Using two complementary datasets—21% O2 (natural conditions) An/Ca curves for four C3 species (Trifolium pratense, Silphium perfoliatum, Lolium perenne, Triticum aestivum) from Ye et al. [26], and our newly collected 2% O2 (photorespiration-suppressed) An/Ca curves for the same species—this study aimed to address three core objectives: (1) Develop a robust analytical framework to extract Rd-related parameters from Aₙ/Cₐ curves under 21% and 2% O2; (2) Evaluate the consistency of 21% O2-derived Rd estimates with 2% O2 data under the assumption that Rₚ is minimized under low O2 (i.e. RC ≈ true Rd), and cross-reference these estimates with literature-derived Rd ranges for C3 crops [1,25,27]. It should be emphasized that this approach provides indirect support rather than independent validation of the RC ≈ Rd assumption. (3) Analyze interspecific differences in Rd stability across O2 levels and link variability to species-specific photosynthetic traits." Specific revisions can be found in Lines 73–84 of the revised manuscript.

Material and methods

Q8• Why 2% O2 is selected as suppressed condition of photorespiration.

Response: We thank the reviewer for this question. The selection of 2% O2 as the photorespiration-suppressed condition was based primarily on the following considerations. First, photorespiration is initiated by the oxygenation reaction of Rubisco, which competes with carboxylation. The rate of oxygenation depends directly on the O2/CO2 ratio at the Rubisco active site. Reducing the O2 concentration from 21% to 2% drastically lowers this ratio, thereby strongly suppressing Rubisco oxygenation and photorespiration. Second, 2% O2 is a widely used condition for suppressing photorespiration in plant physiology studies (e.g., Ku and Edwards, 1977; Brooks and Farquhar, 1985; Busch and Sage, 2017). At this concentration, photorespiration is strongly suppressed without causing severe hypoxia or mitochondrial dysfunction, thus avoiding interference with Rd measurements. Finally, the results of this study also showed that under 2% O2, the CO2 compensation point (Γ) decreased by 50–67% compared with 21% O2, and the gross photorespiratory rate at Ca = 0 (RCO) decreased by 40–55%. These quantitative changes confirm that photorespiration was substantially suppressed under 2% O2.

Reference:

[1] Ku SB, Edwards GE. Oxygen inhibition of photosynthesis: I. Temperature dependence and relation to O2/CO2 solubility ratio. Plant Physiol. 1977; 59: 986–990.

[2] Brooks A, Farquhar GD. Effect of temperature on the CO2/O2 specificity of ribulose‐1,5‐bisphosphate carboxylase/oxygenase and the rate of respiration in the light. Planta. 1985; 165: 397–406.

[3] Busch FA, Sage RF. The sensitivity of photosynthesis to O2 and CO2 concentration identifies strong Rubisco control above the thermal optimum. New Phytol. 2017; 213: 1036–1051.

Q9• Any evidence of minimization of photosynthesis under these conditions.

Response: We thank you for this important question. In the original manuscript, we had already stated that An values under 2% O2 were higher than those under 21% O2 across all four C3 species and all Ca levels (Fig. 2), indicating that photosynthesis was not impaired under low O2. In addition, in the revised manuscript, we have supplemented further evidence showing that photosynthesis was not suppressed under 2% O2. Specifically: (1) Amax increased by approximately 5–25% under 2% O2 compared with 21% O2 across the four C3 species. This increase is consistent with the suppression of photorespiratory carbon loss, which allows a greater proportion of fixed carbon to be retained. (2) No visible symptoms of hypoxia or stress (e.g., leaf chlorosis or necrosis) were observed in any species under 2% O2 during the measurement period. These lines of evidence collectively demonstrate that the 2% O2 treatment suppressed photorespiration without inhibiting photosynthesis, thereby providing a valid approach for isolating Rd. Specific revisions can be found in the Discussion section (Lines 318–324) of the revised manuscript.

Q10• Equation 1, if explained would be better.

Response: This comment has been addressed in the revised manuscript. We have supplemented the derivation of Equation 1 and the meaning of each parameter in the revised manuscript. Specific revisions can be found in Lines 95–96 and 99–105 of the revised manuscript.

Q11• Are these parameters physiologically significant?

Response: The physiological significance of the parameters involved in the model equations has been supplemented in the revised manuscript. Specific revisions can be found in the Materials and Methods section (Lines 99-105) of the revised manuscript. Thank you for your careful review and valuable comments.

Q12• Statistically speaking, increase number of replications.

Response: Thank you for your suggestion. We acknowledge that increasing the number of replications would further enhance the statistical power of this study. Accordingly, in the revised manuscript, we have addressed this concern in the following three aspects. First, in the Materials and Methods section, we have clarified that the measurements of gas exchange and fluorescence data for the four plant species under different O2 concentrations were performed with three independent biological replicates (n = 3), rather than technical replicates. Each biological replicate consisted of independently grown plants under identical conditions, and each An/Ca curve was measured on a separate leaf (See Lines 181–184 of the revised manuscript). Second, although the sample size in this study was relatively small (n = 3), the results were highly consistent across the four species and two O2 treatments, with R² ≥ 0.996 for all model fits and no significant differences between calculated and observed parameter values (p > 0.05). This consistency provides strong evidence for the robustness of our findings (Tables 2 and 3). Finally, we have added a statement at the end of the Discussion section acknowledging that the relatively small sample size (n = 3) is a limitation of this study, and we recommend that future studies increase the number of biological replicates to further validate the method and improve statistical power (See Lines 404–406 of the revised manuscript).

Results

Q13• Don’t interpret biological data in this section.

Response: We thank the reviewer for this suggestion. We have removed all interpretations of biological data from the Results section. For example, we have deleted statements from the original manuscript such as "confirms the strong fitting accuracy of Eqn 1—validating the model's reliability for describing Aₙ responses to Cₐ in C3 plants under varying O2 conditions" and "These patterns confirm that low O2 (2%) suppresses Rc across C3 species, and species-specific respiratory and photosynthetic traits further modulate this response to CO2 and O2 availability." In addition, we have moved the original Section 3.4 to Section 2 (Materials and Methods). Specific revisions can be found in Lines 235, 295-296, and 298–299 of the revised manuscript.

Q14• Compare oxygen treatment and species.

Response: We thank the reviewer for this important suggestion. In the revised manuscript, we have performed statistical comparisons between O2 treatments (21% vs. 2%) and among species. Specific details can be found in Lines 229–235, 266–273, and 286-295 of the revised manuscript.

Discussion

Q15• Compare your results with published work.

Response: Thank you for your constructive suggestion. In the Discussion section of the revised manuscript, we have expanded the comparison of our results with previously published work. Specifically, we have supplemented the comparison of our Rd values with the ranges reported in the literature. Through this detailed comparison, we show that our Rd estimates under both 21% and 2% O2 are generally consistent with previously reported values for C3 crops. Specific revisions can be found in Lines 340–346 of the revised manuscript.

Q16• Discuss limitations, if any.

Response: This comment has been addressed in the revised manuscript. We have added a paragraph at the end of the Discussion section specifically addressing the remaining limitations of this study. We explicitly acknowledge that this study does not provide independent ground-truth measurements of Rd (e.g., via 13C labeling or isotope ratio mass spectrometry), and that the RC ≈ Rd assumption remains a theoretical inference supported by multiple lines of indirect evidence rather than direct proof. We now recommend that future studies validate this approach by combining independent isotope-based measurements on the same leaves. In addition, the sample size (n = 3) represents a further limitation, and increasing the number of biological replicates in future studies would help further validate the method and improve statistical power. Specific revisions can be found in Lines 402–411 of the revised manuscript.

Q17• Discuss how it can be applied in field.

Response: Thank you for your constructive suggestion. In the Discussion section of the revised manuscript, we have added a dedicated subsection discussing the Limitations and Field application of the high-CO2/low-O2 approach. In addition, we have supplemented the optimization directions for field application in the Future Directions of the Conclusion. Specific revisions can be found in the Discussion and Conclusion sections (Lines 412–420 and 442-456) of the revised manuscript.

Figures and Graphs

Q18• Improve figures for readability.

Response: This comment has been addressed in the revised manuscript. We have carefully checked the units of the axes in the figures and the corresponding descriptions in the text to ensure that the units in the text, figures, and tables are unified and correspond one-to-one. Specific revisions can be found in the Results section and the figures.

Conclusion

Q19• Give future perspective for improvement.

Response: This comment has been addressed in the revised manuscript. In the Conclusion section, we have provided future research directions and priorities. In addition, we have supplemented the Limitations of this study in the revised manuscript. Specific revisions can be found in Lines 399–406 of the revised manuscript.

Q20• Highlight study limitations.

Response: This comment has been addressed in the revised manuscript. We have supplemented the Limitations of this study at the end of the Discussion section in the revised manuscript. Specific revisions can be found in Lines 402–411 of the revised manuscript.

References

Q21• Format according to journal style.

Response: We thank the reviewer for pointing out the reference formatting issue. We have thoroughly checked and uniformly revised the references according to the Plos One journal style. Specifically, we have standardized the abbreviation format of author names, the abbreviation conventions for journal titles, and the presentation of volume and page numbers. The revised references have been checked item by item against the journal's submission guidelines to ensure full compliance. Specific revisions can be found in the References section of the revised manuscript.

Overall recommendations

Q22• Thoroughly check for English grammar correction and typos.

Response: We thank you for this suggestion. We have thoroughly checked the entire manuscript for English grammar, spelling, and typographical errors. All co-authors have carefully proofread the manuscript multiple times; in addition, we have invited a native English-speaking expert to comprehensively check and revise the English grammar throughout the manuscript; furthermore, we have conducted a thorough check for consistency in terminology, abbreviations, and formatting throughout the manuscript. Specific revisions can be found in the red-highlighted parts of the revised manuscript.

Q23• Recheck the abbreviations.

Response: This comment has been addressed in the revised manuscript. We have checked and verified all abbreviations throughout the manuscript (including those in the text, figures, and tables), and have supplemented and revised Table 1 (the list of abbreviations).

Reviewer #2:

Q1The statistical data has been given in detail and written in an intelligible fashion. The manuscript is sound and the authors have made the findings available. The statistical data has been given in detail for the readers to understand the leaf day respiration.

Response: We sincerely thank you for the positive and encouraging assessment of our manuscript. We are pleased that you found the statistical data to be presented in detail and in an intelligible fashion, and that the manuscript was considered sound with findings made available to readers. We are particularly grateful for your recognition that the statistical data we provided is sufficient to help readers understand leaf day respiration. This aligns with our goal of ensuring transparency and reproducibility in our analysis. In the revised manuscript, we have carefully proofread and revised the manuscript. Thank you for your time and valuable comments.

Reviewer #3:

The manuscript addresses an important and difficult problem in leaf gas-exchange physiology, with the four-species, two O2 - level dataset is a quite reasonable empirical foundation but in its current form the manuscript has three major weaknesses that does not support the central claim that this is a “validated” method for isolating Rd from Rp.

Response: We sincerely thank the reviewer for identifying these critical issues, which have helped us substantially improve the rigor and transparency of our manuscript. In response to the issues raised, we have addressed and replied to each point in the revised manuscript.

Q11. The validation logic is circular i.e. Eqn 1 reproducing its own input data, is repeatedly presented as if it validates the biological assumption that RC approximates Rd under the chosen conditions. No independent ground - reality measurement on the same leaves is presented.

Response: We fully agree with the reviewer that fitting Eqn 1 to the same Aₙ/Cₐ data used for parameter extraction does not constitute independent validation of the biological assumption that RC ≈ Rd under 2% O2. To address this concern, we have made the following revisions in the revised manuscript:

(1) Revised the wording to clearly distinguish between "model goodness-of-fit" and "biological validation": In the revised manuscript, we have carefully distinguished between the goodness-of-fit of Eqn 1 to the Aₙ/Cₐ data and the biological validity of the RC ≈ Rd assumption. We now explicitly state that the high R2 values only demonstrate that Eqn 1 adequately describes the Aₙ/Cₐ response and do not by themselves validate the assumption that photorespiration is suppressed under 2% O2.

(2) Added independent lines of evidence to support the RC ≈ Rd assumption: First, at 2% O2, the O2/CO2 ratio in the chloroplast is drastically reduced, and Rubisco oxygenation is thermodynamically and kinetically suppressed. This is well-established in the literature (e.g., Ku and Edwards, 1977; Brooks and Farquhar, 1985). Second, the CO2 compensation point (Γ) under 2% O2 decreased by 50–67% compared with 21% O2, consistent with the expected suppression of photorespiration. If Rp were not suppressed, Γ would not decrease to this extent. Finally, regarding consistency with literature values: the Rd estimates derived from our 2% O2 data (0.74–0.94 μmol m⁻2 s⁻1) fall within the range reported in the literature for C3 crops (0.52–1.75 μmol m⁻2 s⁻1 at 21% O2; 0.74–2.89 μmol m⁻2 s⁻1 at 2% O2; Yin et al., 2011; Tcherkez et al., 2017).

(3) Explicitly acknowledged the limitations of validation in the Discussion: We have added a paragraph at the end of the Discussion section explicitly acknowledging that our study does not provide independent ground-truth measurements of Rd (e.g., via 13C labeling or isotope ratio mass spectrometry), and that the RC ≈ Rd assumption remains a theoretical inference supported by multiple lines of indirect evidence rather than direct proof. We now recommend that future studies validate this approach by combining independent isotope-based measurements of Rd on the same leaves.

Specific revisions can be found in the Abstract, Materials and Methods, and Discussion sections (Lines 35-37; 166-169; 327-333; 402–404) of the revised manuscript. We appreciate your valuable comments.

Reference:

[1] Ku, S.B.; Edwards, G.E. Oxygen inhibition of photosynthesis: I. Temperature dependence and relation to O2/CO2 solubility ratio. Plant Physiol. 1977, 59, 986-990.

[2] Brooks, A.; Farquhar, G.D. Effect of temperature on the CO2/O2 specificity of ribulose‐1,5‐bisphosphate carboxylase/oxygenase and the rate of respiration in the light. Planta. 1985, 165, 397–406.

[3] Yin, X.Y.; Sun, Z.P.; Struik, P.C.; Gu, J.F. Evaluating a new method to estimate the rate of leaf respiration in the light by analysis of combined gas exchange and chlorophyll fluorescence measurements. J. Exp. Bot. 2011, 62, 3489–3499.

[4] Tcherkez, G.; Gauthier, P.; Buckley, T.N.; Busch, F.A.; Barbour, M.M.; Bruhn, D.; Heskel, M.A.; Gong, X.Y.; Crous, K.Y.; Griffin, K.; Way, D.; Turnbull, M.; Adams, M.A.; Atkin, O.K.; Farquhar, G.D.; Cornic, G. Leaf day respiration: low CO2 flux but high significance for metabolism and carbon balance. New Phytol. 2017, 216, 986–1001.

Q22. The results are internally inconsistent i.e. unit assignments in the model are not dimensionally coherent, Figures 2 and 3 display units that contradict the surrounding text and tables, and three different Rd ranges are reported for what should be a single caption number.

Response: We thank the reviewer for pointing out these inconsistencies. We have carefully re-examined all units, figures, tables, and numerical values throughout the manuscript and have made the following corrections:

(1) An (unit: mmol m–2 s–1); Ca (unit: mmol mol–1); Γ (unit: mmol mol–1); � (unit: mol m–2 s–1, after multiplying by Ca in μmol mol–1, the units become mmol m–2 s–1); � (unit: mol μmol–1, dimensionless when multiplied by Ca in μmol mol–1); � (unit: mol mmol–1, , dimensionless when multiplied by Ca in μmol mol–1). We have added a unit consistency check in the Table 1 and Materials and Methods (Section 2.1) to clarify this comment.

(2) Regarding the inconsistency between the units of the coordinate axes in Figures 2 and 3 and the units in the text and tables, after verification, we found that there was an error in the units when we used Origin to draw the output. The same issue also exists in Figure 1. The coordinate axis units in Figures 1, 2, and 3 have been modified in the revised manuscript. Thank you again for your careful review.

(3) We sincerely apologize for the inconsistency in reporting the Rd ranges. We have now carefully reviewed all instances where Rd values are reported and have unified them. For example, in the Abstract, according to Table 4, the estimated Rd values under high-CO2/low-O2 (>1200 μmol mol–1 CO2 + 2% O2) conditions range from 0.74 to 1.12 μmol m–2 s–1. This falls within the range of 0.74–2.89 μmol m–2 s⁻1 reported under 2% O2 in the literature we cited Yin et al. [2]. In the original manuscript, we cited a different reference in the Abstract, which led to the inconsistency in the reported Rd range. We have now unified this in the revised manuscript. In addition, we have also supplemented the Results section with a statement that the estimated Rd values under high- CO2/low-O2 (>1200 μmol mol–1 CO2 + 2% O2) conditions range from 0.74 to 1.12 μmol m–2 s–1. Thank you for your careful review.

Q33. Main experimental controls are unverified as the actual O2 concentration delivered by the buffer - bag system over the course of a measurement is not reported, despite this being the essential operation of entire study.

Response: We fully agree that verifying the actual O2 concentration delivered to the leaf chamber is essential for the credibility of our study. In this study, gas exchange and fluorescence data were measured using a LI-6400 portable photosynthesis system. The LI-6400 is an open-path infrared gas analysis system designed primarily for measuring CO2 and H2O concentration changes. O2 concentration is not included in the built-in measurement or display parameters of the LI-6400. Therefore, for the 2% O2 treatment in this study, an external gas supply approach was adopted: a pre-prepared low-oxygen gas cylinder (e.g., "The 2% O2 gas mixture was purchased from Ouyuan Gas Plant (Wenzhou, Zhejiang) with a certified composition of 2% O2 + 98% N2.") was first injected into a self-made gas buffer bag containing a certain amount of water to mimic atmospheric humidity, and then connected to the LI-6400 inlet via a connector. This measurement method has been granted a Chinese National Utility Model Patent (Patent No.: ZL 2015 2 0174847.1). Based on this method, we have conducted related research for over ten years and have published multiple papers involving this technical approach (Kang et al., 2014; Niu et al., 2025; Ye et al., 2025).

Reference:

[1] Ye, Z.P.; Yang, X.L.; Ye, Z.W.Y.; An, T.; Duan, S.H.; Kang, H.J.; Wang, F.B. Evaluating photosynthetic models and their potency in assessing plant responses to changing oxygen concentrations: a comparative analysis of An–Ca and An–Ci curves in Lolium perenne and Triticum aestivum. Front. Plant Sci. 2025, 16, 1575217.

[2] Niu, Z.W.; Ye, Z.W.Y.; Huang, Q.; Peng, C.; Kang, H.J. Accuracy of photorespiration and mitochondrial respiration in the light fitted by CO2 response model for photosynthesis. Front. Plant Sci. 2025, 16, 1455533.

[3] Kang, H.J.; Tao, Y.L.; Quan, W.; Wang, W.; OuYang, Z. Fitting mitochondrial respiration rates under light by photosynthetic CO2 response models. Chin J Plant Ecol. 2014, 38(12), 1356-1363.

Reviewer #4:

The study on very important and applied aspect of plant physiology has been performed with standard protocols and it sounds technically excellent. The conclusions drwan are very relevent to data presented in text, tables and figures. It has been supported with future prospects of the study. The data generated has been rigorously and statistically analysed using latest models and softwares. And the data is visible in tabulted as well as figures form and no part is hiddden. The flow and sequence of the whole manuscript is very meaningful and self explanatory. The subheadinds explaining different aspects and parametres related to photosynthesis, photorespiration and day respiration are impressive in results as well as in discussion section. The sequence, placement and explanations of mathematical equations is excellent. Specially the explanations of results of application of new model for separating day resiration from photorespiration is very impressive. All this has been narrated in standard English language without any difficulty to understand by non english persons as well....Overall this MS has revealed understanding of future Co2 concentrations and its impact calculation for C3 species which are prone to photorespiration.

Response: We sincerely thank the reviewer for the highly positive and encouraging evaluation of our manuscript. We are delighted that the reviewer found our study to address an important and applied aspect of plant physiology, and that the experimental protocols, statistical analyses, data presentation, and overall structure were considered technically excellent and meaningful. We are particularly grateful for the reviewer's recognition of:

(1) The relevance and consistency of our conclusions with the data presented in the text, tables, and figures;

(2) The rigorous statistical analysis using up-to-date models and software;

(3) The clarity and transparency of the data, with no part hidden;

(4) The logical flow and self-explanatory structure of the manuscript;

(5) The impressive presentation and explanation of mathematical equations, especially those related to the new model for separating day respiration from photorespiration;

(6) The standard and accessible English language used throughout.

We also appreciate the reviewer's acknowledgment that our study contributes to understanding future CO2 concentrations and their impact on C3 species prone to photorespiration. This encouragement strongly motivates our continued work in this area.

We have carefully reviewed the manuscript once more. All co-authors have read and approved the final version. Thank you again for your time and valuable comments.

Sincerely,

Fubiao-Wang

The corresponding autho

# Peer review: Decision Letter 1

**DOI:** 10.1371/journal.pone.0361238.r003

**Authors:** Thomas Roach

8 Oct 2026

Disentangling Leaf Day Respiration from Photorespiration in C3 Leaves: A Single-Curve An/Ca Approach Validated under Photorespiration-Suppressed Conditions

PONE-D-26-26924R1

Dear Dr. Wang,

We’re pleased to inform you that your manuscript has been judged scientifically suitable for publication and will be formally accepted for publication once it meets all outstanding technical requirements.

Within one week, you’ll receive an e-mail detailing the required amendments. When these have been addressed, you’ll receive a formal acceptance letter and your manuscript will be scheduled for publication.

An invoice will be generated when your article is formally accepted. Please note, if your institution has a publishing partnership with PLOS and your article meets the relevant criteria, all or part of your publication costs will be covered. Please make sure your user information is up-to-date by logging into Editorial Manager at Editorial Manager® and clicking the ‘Update My Information' link at the top of the page. For questions related to billing, please contact billing support.

If your institution or institutions have a press office, please notify them about your upcoming paper to help maximize its impact. If they’ll be preparing press materials, please inform our press team as soon as possible -- no later than 48 hours after receiving the formal acceptance. Your manuscript will remain under strict press embargo until 2 pm Eastern Time on the date of publication. For more information, please contact onepress@plos.org.

Kind regards,

Thomas Roach

Academic Editor

PLOS One

Additional Editor Comments (optional):

Reviewers' comments:

Reviewer's Responses to Questions

**Comments to the Author**

Reviewer #1: All comments have been addressed

**********

2. Is the manuscript technically sound, and do the data support the conclusions?

Reviewer #1: Yes

**********

3. Has the statistical analysis been performed appropriately and rigorously?

Reviewer #1: Yes

**********

4. Have the authors made all data underlying the findings in their manuscript fully available?

The PLOS Data policy

Reviewer #1: Yes

**********

5. Is the manuscript presented in an intelligible fashion and written in standard English?

Reviewer #1: Yes

**********

Reviewer #1: (No Response)

**********

what does this mean?). If published, this will include your full peer review and any attached files.

If you choose “no”, your identity will remain anonymous but your review may still be made public.

**Do you want your identity to be public for this peer review?**  For information about this choice, including consent withdrawal, please see our Privacy Policy

Reviewer #1: **Yes:**  Dr. Syed Hassan Ali Shah

**********

# Editor report: Acceptance letter

**DOI:** 10.1371/journal.pone.0361238.r004

**Authors:** Thomas Roach

PONE-D-26-26924R1

PLOS One

Dear Dr. Wang,

I'm pleased to inform you that your manuscript has been deemed suitable for publication in PLOS One. Congratulations! Your manuscript is now being handed over to our production team.

At this stage, our production department will prepare your paper for publication. This includes ensuring the following:

* All references, tables, and figures are properly cited

* All relevant supporting information is included in the manuscript submission,

* There are no issues that prevent the paper from being properly typeset

You will receive further instructions from the production team, including instructions on how to review your proof when it is ready. Please keep in mind that we are working through a large volume of accepted articles, so please give us a few days to review your paper and let you know the next and final steps.

Lastly, if your institution or institutions have a press office, please let them know about your upcoming paper now to help maximize its impact. If they'll be preparing press materials, please inform our press team within the next 48 hours. Your manuscript will remain under strict press embargo until 2 pm Eastern Time on the date of publication. For more information, please contact onepress@plos.org.

You will receive an invoice from PLOS for your publication fee after your manuscript has reached the completed accept phase. If you receive an email requesting payment before acceptance or for any other service, this may be a phishing scheme. Learn how to identify phishing emails and protect your accounts at https://explore.plos.org/phishing.

If we can help with anything else, please email us at customercare@plos.org.

Thank you for submitting your work to PLOS One and supporting open access.

Kind regards,

PLOS One Editorial Office Staff

on behalf of

Dr. Thomas Roach

Academic Editor

PLOS One
